# Strictly regulated agonist-dependent activation of AMPA-R is the key characteristic of TAK-653 for robust synaptic responses and cognitive improvement

**DOI:** 10.1038/s41598-021-93888-0

**Published:** 2021-07-15

**Authors:** Atsushi Suzuki, Akiyoshi Kunugi, Yasukazu Tajima, Noriko Suzuki, Motohisa Suzuki, Masashi Toyofuku, Haruhiko Kuno, Satoshi Sogabe, Yohei Kosugi, Yasuyuki Awasaki, Tomohiro Kaku, Haruhide Kimura

**Affiliations:** 1 Neuroscience Drug Discovery Unit, Research, Takeda Pharmaceutical Company Limited, 26-1, Muraoka-Higashi 2-chome, Kanagawa 251-8555 Fujisawa, Japan; 2 Bio-Molecular Research Laboratories, Research, Takeda Pharmaceutical Company Limited, Fujisawa, Japan; 3 Drug Metabolism and Pharmacokinetics Research Laboratories, Research, Takeda Pharmaceutical Company Limited, Fujisawa, Japan; 4 Drug Safety Research and Evaluation, Research, Takeda Pharmaceutical Company Limited, Fujisawa, Japan

**Keywords:** Ligand-gated ion channels, Pharmacology, Drug discovery, Target validation

## Abstract

Agonistic profiles of AMPA receptor (AMPA-R) potentiators may be associated with seizure risk and bell-shaped dose-response effects. Here, we report the pharmacological characteristics of a novel AMPA-R potentiator, TAK-653, which exhibits minimal agonistic properties. TAK-653 bound to the ligand binding domain of recombinant AMPA-R in a glutamate-dependent manner. TAK-653 strictly potentiated a glutamate-induced Ca^2+^ influx in hGluA1i-expressing CHO cells through structural interference at Ser743 in GluA1. In primary neurons, TAK-653 augmented AMPA-induced Ca^2+^ influx and AMPA-elicited currents via physiological AMPA-R with little agonistic effects. Interestingly, TAK-653 enhanced electrically evoked AMPA-R-mediated EPSPs more potently than AMPA (agonist) or LY451646 (AMPA-R potentiator with a prominent agonistic effect) in brain slices. Moreover, TAK-653 improved cognition for both working memory and recognition memory, while LY451646 did so only for recognition memory, and AMPA did not improve either. These data suggest that the facilitation of phasic AMPA-R activation by physiologically-released glutamate is the key to enhancing synaptic and cognitive functions, and nonselective activation of resting AMPA-Rs may negatively affect this process. Importantly, TAK-653 had a wide safety margin against convulsion; TAK-653 showed a 419-fold (plasma C_max_) and 1017-fold (AUC _plasma_) margin in rats. These findings provide insight into a therapeutically important aspect of AMPA-R potentiation.

## Introduction

Glutamate is the primary excitatory neurotransmitter throughout the central nervous system (CNS). Enhancement of glutamate-mediated transmission is critical for synaptic plasticity and for learning and memory^1–3^. Glutamatergic dysfunction has been implicated in various disorders including schizophrenia, Alzheimer’s disease (AD), attention-deficit/hyperactivity disorder (ADHD), autism and major depressive disorders; thus, potentiation of glutamatergic transmission could be a promising therapeutic strategy for psychiatric and neurological diseases^4–6^.

Multiple lines of evidence support activation of the alpha-amino-3-hydroxy-5-methyl-4-isoxazole-propionic acid receptor (AMPA-R) as a promising strategy for the treatment of CNS disorders^7–11^. However, AMPA-R agonists have a high seizure risk probably due to nonspecific activation of resting AMPA-R in the brain^12–15^, and hence are limited in their ability to secure a safety margin. As glutamate release in the brain is strictly regulated, AMPA-R potentiators (= AMPA-R positive allosteric modulators) which can promote physiological AMPA-R activation by glutamate have been considered as an alternative approach. However, AMPA-R potentiators such as LY451646 showed prominent agonistic effects (AMPA-R activation in the absence of agonist) in Ca^2^^+^ influx assays and whole-cell current recordings using primary hippocampal neurons^16^. Moreover, LY451646 had a narrow safety margin against seizures and a narrow bell-shaped dose-response for cognitive improvement in rats and monkeys^16^. A bell-shaped dose-response was also seen in various other pharmacological assays of the AMPA-R potentiators LY451646 and S18986^17–19^. The bell-shaped dose-response of AMPA-R potentiators may be due to desensitization of AMPA-R; however, this hypothesis is inconsistent with their seizure induction at higher doses. Thus, further characterization of the mechanisms of action underlying the bell-shaped dose-response effect of AMPA-R potentiators is needed.

We recently reported that structural interference at Ser750 in AMPA-R GluA2o (corresponding to Ser743 in GluA1i) was key to lowering the agonistic effect of AMPA-R potentiators containing a dihydropyridothiadiazine 2,2-dioxides skeleton^16^. To discover novel AMPA-R potentiators with better pharmacological profiles, we established an original screening strategy consisting of 1) binding assays with or without glutamate using [^3^H]-HBT1 and purified recombinant ligand-binding domain (LBD) of AMPA-R, 2) X-ray crystallography, 3) Ca^2+^ influx assays using wild-type hGluA1i or hGluA1i with a mutation at Ser743 in CHO cells, 4) Ca^2+^ influx assay in rat primary hippocampal neurons, and 5) whole-cell patch clamp recording using rat primary hippocampal neurons. By using the screening strategy, we designed and searched for AMPA-R potentiators with lower agonistic effects and discovered TAK-137. TAK-137 bound to AMPA-R in a glutamate-dependent manner and showed a lower agonistic effect than LY451646 in both Ca^2+^ influx assay and whole-cell patch clamp recording in rat primary hippocampal neurons. Studies in rats found potent cognitive improvement and a wider safety margin against seizures with TAK-137 as compared with LY451646. However, TAK-137 still maintained a weak agonistic effect^16,20^.

AMPA-R potentiators with lower agonistic effects than TAK-137 have not been explored yet; such a compound may have a wider safety margin against seizures or may lose beneficial effects, including cognitive improvement. Thus, we decided to discover and characterize a novel AMPA-R potentiator with lower agonistic effects than TAK-137. Through our original screening strategy, we discovered TAK-653, 9-[4-(Cyclohexyloxy)phenyl]-7-methyl-3,4-dihydropyrazino[2,1-c][1,2,4]thiadiazine 2,2-dioxide, which had virtually no agonistic effects in primary hippocampal neurons. Here, we describe the pharmacological characteristics of TAK-653. Our data suggest that the facilitation of phasic AMPA-R activation by physiologically-released endogenous agonist (glutamate) is the key to enhance synaptic and cognitive functions, and nonselective activation of resting AMPA-Rs by agonistic effects may negatively affect both synaptic and cognitive functions, resulting in bell-shaped dose-response effects.

## Results

### TAK-653 bound to the LBD of purified recombinant AMPA-R proteins and induced Ca^2+^ influx in hGluA1i CHO cells in a glutamate-dependent manner

**Figure 1:**
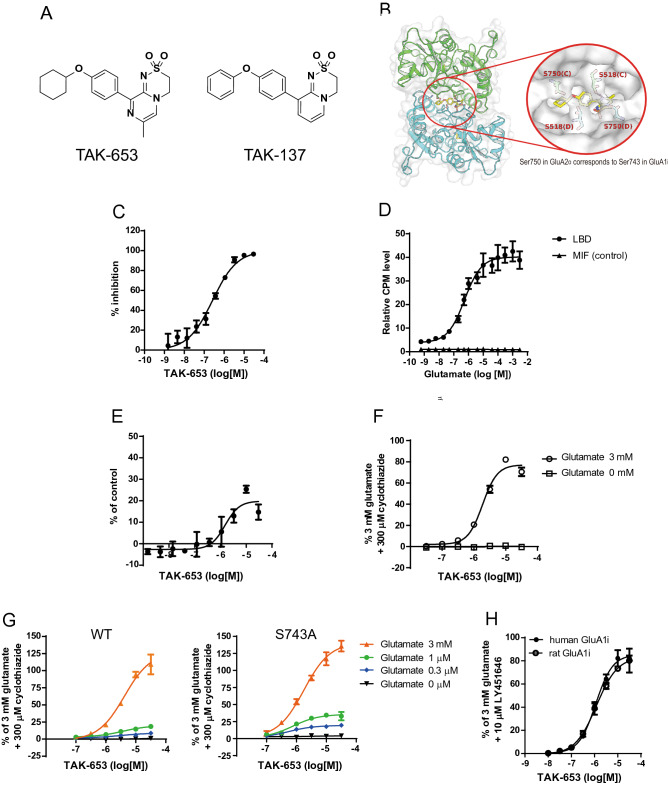
Selective binding of TAK-653 to AMPA-R in a glutamate-dependent manner. (**A**) Chemical structure of TAK-653 and TAK-137. (**B**) The GluA2 (flop)/glutamate/TAK-653 complex crystallized as a dimer. (**C**) Displacement studies with TAK-653 using the SPA assay with [^3^H]-HBT1 and His-LBD. Data are represented as mean ± SD (n = 4). (**D**) Effects of glutamate on the binding of [^3^H]-TAK-653 to His-LBD. Data are represented as relative value ± SD in counts per minute (CPM) (n = 3). (**E**) Effect of TAK-653 on the binding of [^3^H]-AMPA to His-LBD. Data are represented as mean ± SEM (n = 4). (**F**) Effects of TAK-653 on Ca^2+^ influx in GluA1i CHO cells in the presence (open circle) and absence (open square) of 3 mM glutamate. Data are represented as mean ± SD (n = 3). (**G**) Effects of TAK-653 on Ca^2+^ influx in CHO cells expressing GluA1i WT or GluA1i S743A in the absence or presence of glutamate (0.3 μM, 1 μM and 3 mM). S743 in GluA1i LBD corresponds to S750 in GluA2o LBD. Data are represented as mean ± SD (n = 3). (**H**) Effects of TAK-653 on Ca^2+^ influx in hGluA1i CHO cells (closed circle) and rGluA1i CHO cells (open circle) in the presence of 3 mM glutamate. Data are represented as mean ± SD (n = 3).

We previously reported that structural interference at Ser750 in the channel-closed state of GluA2o LBD might be involved in the molecular mechanisms underlying the lower agonistic effect of AMPA-R potentiators with dihydropyridothiadiazine 2,2-dioxide derivatives and discovered TAK-137, 9-(4-phenoxyphenyl)-3,4-dihydropyrido[2,1-c][1,2,4]thiadiazine 2,2-dioxide (Fig. 1A)^16^. Based on the structure and agonistic-effect relationship studies on this novel chemical series, we designed TAK-653, a dihydropyrazinothiadiazine 2,2-dioxide derivative with cyclohexyl group as bulky terminal substituents to induce steric repulsion at Ser750 in GluA2o; the structural bulkiness of cyclohexyl group is higher than that of the terminal phenyl group of TAK-137, suggesting that TAK-653 may exhibit a lower agonistic effect. TAK-653 bound to the intradimer interface formed by the ligand binding core (Fig. 1B). The 3D overlay analysis showed that the peripheral cyclohexyl rings of TAK-653 caused steric interference at Ser750 in the channel-closed state of GluA2o LBD (Toyofuku et al., in preparation). Binding affinity of TAK-653 to the GluA2o LBD was measured by a scintillation proximity assay (SPA) using [^3^H]-HBT1, a radio-labeled LBD-binding AMPA-R potentiator, and a His-tagged GluA2o LBD protein (His-LBD)^21^. TAK-653 inhibited binding between [^3^H]-HBT1 and His-LBD with an IC_50_ value of 0.26 μM (Fig. 1C). Binding of [^3^H]-TAK-653 to His-LBD was also measured by SPA. Binding between [^3^H]-TAK-653 and His-LBD was robustly increased in a glutamate-dependent manner, whereas binding of [^3^H]-TAK-653 was not detected when another His-tagged protein such as macrophage migration inhibitory factor (MIF) was used as a control (Fig. 1D). TAK-653 did not inhibit the specific binding of [^3^H]-AMPA to His-LBD, but rather mildly increased [^3^H]-AMPA binding with EC_50_ and E_max_ values of 1.5 ± 0.2 μM and 19.9 ± 3.4%, respectively (Fig. 1E), suggesting that TAK-653 has no binding affinity for the agonist binding site of AMPA-R. These results suggest that TAK-653 selectively binds to the LBD of AMPA-R in a glutamate-dependent manner due to structural interference at Ser750 (GluA2o LBD) in the channel-closed state.

In the functional assays, TAK-653 robustly increased Ca^2+^ influx only in the presence of glutamate (3 mM) in hGluA1i CHO cells; the EC_50_ was 3.3 μM (Fig. 1F). Ser750 in GluA2o LBD corresponds to Ser743 in GluA1i LBD, thus the introduction of an S743A mutation into GluA1i was expected to reduce the steric interference with the peripheral cyclohexyl rings of TAK-653 and to facilitate the binding of TAK-653 to GluA1i especially when glutamate concentration is low. In fact, the maximum responses of TAK-653 in CHO cells expressing S743A GluA1i were higher than those in CHO cells expressing wild-type GluA1i in a Ca^2+^ influx assay at each concentration of glutamate tested (Fig. 1G). Next, to improve the prediction of efficacious concentration for use in humans, we assessed potential of TAK-653 in human and rat GluA1i. In a Ca^2+^ influx assay using human and rat GluA1i-expressing CHO cells, TAK-653 did not show species differences; the fold difference in the EC_50_ value between rat and human receptors was 1.1 (Fig. 1H). TAK-653 did not show prominent subunit selectivity for homomeric AMPA-R in a Ca^2+^ influx assay using CHO cells expressing GluA1-4i and TARP γ-2 or GluA1-4o and TARP γ-2 (Table S1). TAK-653 at 10 μM was highly selective against 97 targets (Ricerca, Taipei, Taiwan); TAK-653 only inhibited lipoxygenase 5-LO enzyme activity with an IC_50_ value of 5.9 μM (Table S2). These observations further suggest that TAK-653 selectively binds to AMPA-R in a glutamate-dependent manner due to structural interference at Ser750 in GluA2o LBD (S743A in GluA1i LBD).

### TAK-653 induced Ca^2+^ influx, whole-cell currents, and brain-derived neurotrophic factor (BDNF) production in an AMPA-dependent manner in primary hippocampal neurons

**Figure 2:**
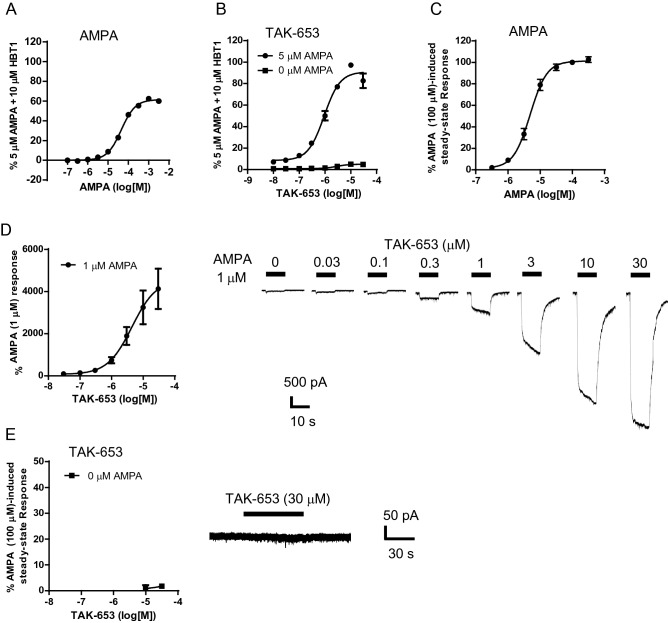
Effect of TAK-653 on Ca^2+^ influx and AMPA-R currents in rat primary hippocampal neurons. (**A**) Effect of AMPA on Ca^2+^ influx in primary hippocampal neurons. (**B**) Effect of TAK-653 on Ca^2+^ influx in primary hippocampal neurons. TAK-653 was applied in the presence or absence of 5 μM AMPA. Data are represented as mean ± SD (n = 3). (**C**) Effects of AMPA on AMPA-R-mediated currents using primary hippocampal neurons. (**D**, **E**) Effects of TAK-653 on AMPA-R-mediated currents using primary hippocampal neurons. The responses to TAK-653 were measured in the presence (**D**) or absence (**E**) of 1 μM AMPA. AMPA-R potentiator was applied 20 s prior to 10-s AMPA stimulus. Without AMPA, the maximum current was measured during 60-s application. Data are represented as mean ± SEM (n = 6).

In our previous studies, recombinant AMPA-R on hGluA1i CHO cells was less sensitive than physiological AMPA-R on primary neurons in the detection of agonistic effects of AMPA-R potentiators^16,21^. Thus, we next characterized TAK-653 using rat primary hippocampal neurons. A single application of AMPA dose-dependently increased intracellular Ca^2+^ levels (Fig. 2A). Thus, our experimental conditions are suitable for the characterization of AMPA-R potentiators using cultured primary neurons. The response of 5 μM AMPA plus 10 μM HBT1 was defined as 100% in the Ca^2+^ influx assay using rat primary hippocampal neurons^21^. TAK-653 robustly increased Ca^2+^ influx only in the presence of AMPA (5 μM) with an EC_50_ of 0.93 μM (Fig. 2B). TAK-653, TAK-137, and LY451646 at 30 μM achieved the maximal Ca^2+^ increase in the presence of agonist (AMPA), thus the response at 30 μM was used to compare their agonistic effects. The Ca^2+^ increase produced by TAK-653, TAK-137, and LY451646 at 30 μM was 4.8%, 7.6%, and 88%, respectively (Table 1)^16^.

**Table 1:** Effects on intracellular Ca^2+^ level and AMPA-R-mediated currents in the presence or absence of AMPA in rat primary hippocampal neurons.

	Intracellular Ca^2+^ level *		AMPA-R currents ^#^	
	Potentiation EC_50_ (μM)	Agonistic effect @30 μM (%)	Potentiation EC_50_ (μM)	Agonistic effect @30 μM (%)
TAK-653	0.93	4.8	4.4	1.7
TAK-137	0.42	7.6	1.4	6.4
LY451646	0.78	88	1.9	39

*% of 5 μM AMPA + 10 μM HBT1 response.

^#^ % of AMPA (100 μM)-induced steady-state response.

EC_50_ value was calculated from dose-response curve in the presence of 5 μM AMPA for intracellular Ca^2+^ levels or 1 μM AMPA for AMPA-R currents using a nonlinear regression.

In whole-cell patch clamp recordings using rat primary hippocampal neurons, a single application of AMPA dose-dependently increased AMPA-R-mediated currents with an EC_50_ value of 45 μM (Fig. 2C). Thus, AMPA at a low dose of 1 μM was used to characterize the AMPA-R potentiation activity of TAK-653. The response of TAK-653 with AMPA (1 μM) was normalized against currents induced by 1 μM AMPA. TAK-653 dose-dependently augmented AMPA (1 μM)-elicited currents with an EC_50_ value of 4.4 μM (Fig. 2D). Next, we assessed agonistic effect of TAK-653. In the absence of agonist, the response of TAK-653 was normalized against currents induced by 100 μM AMPA. As a result, TAK-653 even at 30 μM produced 1.7% of the AMPA (100 μM)-elicited currents, while TAK-137 and LY451646 showed 6.4% and 39%, respectively, response at 30 μM (Fig. 2E and Table 1)^16,20^. Thus, the agonistic effects of TAK-653 were lower than those of TAK-137 and LY451646 in rat primary hippocampal neurons.

**Figure 3:**
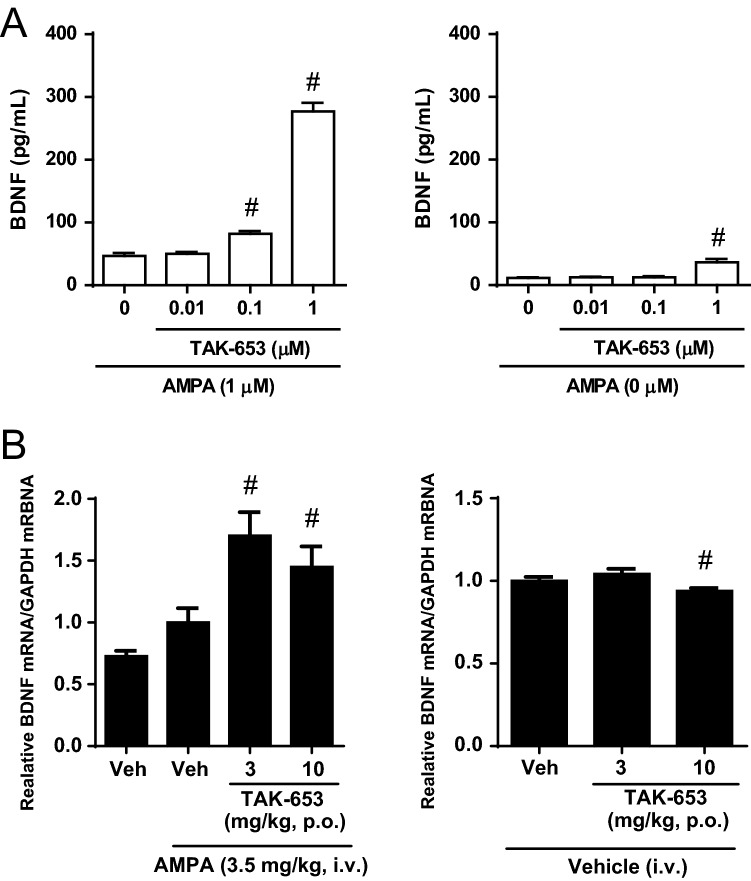
Effects of TAK-653 on BDNF expression in vitro and in vivo. (**A**) Effects of TAK-653 on BDNF protein levels in rat primary hippocampal neurons. Cells were treated with AMPA (0 or 1 μM) and TAK-653 (0.01, 0.1, 1 μM) for 24 h and then were collected using lysis buffer. Cells in control group were treated with 1 μM AMPA and DMSO. Values were expressed as pg per mL. Data are represented as mean ± SD (n = 3). Statistical significance was determined by a two-tailed Williams’ test with significance set at ^#^*P* ≤ 0.05 (versus control group; two-tailed Williams’ test). (**B**) Effects of TAK-653 on BDNF mRNA in hippocampus in AMPA (3.5 mg/kg, i.v.)-treated mice. TAK-653 (3 and 10 mg/kg, p.o.) was administered to mice 1 h before the administration of AMPA (3.5 mg/kg, i.v.) (left) or vehicle (right). Tissues were isolated 3 h after AMPA administration. Data were presented as the mean ± SEM (n = 23–24). ^#^*P* ≤ 0.05 (versus vehicle-treated group; two-tailed Williams’ test).

Activation of AMPA-R increased BDNF mRNA levels in the mouse hippocampus^22,23^. We examined the effect of TAK-653 on BDNF production in rat primary hippocampal neurons. TAK-653 robustly increased BDNF protein levels in the presence of a low concentration of AMPA (1 μM) (Fig. 3A). TAK-653 alone slightly increased BDNF protein levels at 1 μM. Next, we investigated the effect of TAK-653 on BDNF mRNA levels in the mouse hippocampus. TAK-653 at 3 and 10 mg/kg, p.o. significantly increased BDNF mRNA levels in the AMPA (3.5 mg/kg, i.v.)-treated mice, while TAK-653 alone did not increase BDNF mRNA levels under these experimental conditions (Fig. 3B). These results demonstrate that TAK-653 stimulates BDNF production through agonist-dependent AMPA-R activation in both in vitro and in vivo.

### TAK-653, but not LY451646 or AMPA, improved cognitive functions in multiple domains in rats and working memory in monkeys

**Figure 4:**
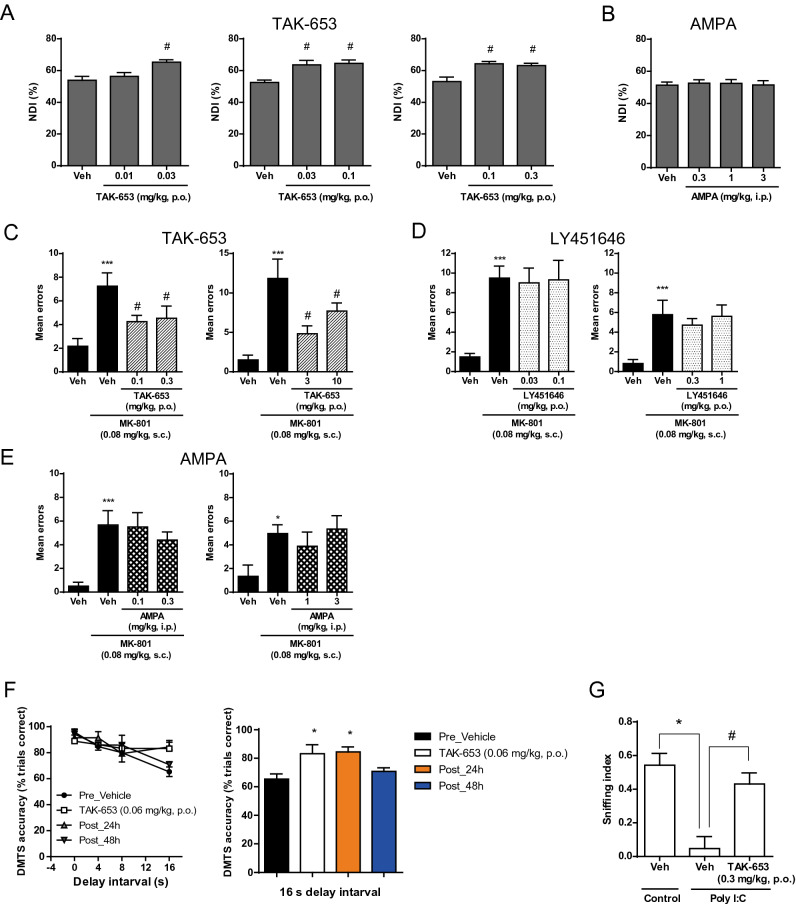
Effect of TAK-653, LY451646, and AMPA on cognitive functions in multiple domains. (**A**) Effect of TAK-653 on visual learning and memory in NOR test using naïve rats. TAK-653 (0.01 and 0.03 mg/kg, p.o., 0.03 and 0.1 mg/kg, p.o., and 0.1 and 0.3 mg/kg, p.o.) was administered to rats 2 h prior to the acquisition and the retention trials. (**B**) Effect of AMPA on visual learning and memory in NOR test using naïve rats. AMPA (0.3, 1 and 3 mg/kg, i.p.) was administered 0.5 h prior to the acquisition and the retention trials. NDI data were presented as the mean ± SEM (n = 10). ^#^*P* ≤ 0.05 (versus vehicle-treated group; two-tailed Williams’ test). (**C**–**E**) Effect of TAK-653 (**C**), LY451646 (**D**) or AMPA (**E**) on working memory in RAM test using rats. At 1.5 h, 1 h or 0 h before administration of vehicle or MK-801 (0.08 mg/kg, s.c.), TAK-653, LY451646 or AMPA, respectively, was administered to rats. Thirty minutes after dosing of MK-801, rats were placed on the maze, and then the entry into the arm was recorded. The mean errors were indicated as the mean ± SEM (n = 6–18). ***P ≤ 0.001 (versus vehicle-vehicle group; Welch’s test); ^#^*P* ≤ 0.05 (versus vehicle-MK-801 group; two-tailed Shirley-Williams’ test). (**F**) Effect of TAK-653 on working memory in DMTS test using monkeys. TAK-653 at 0.06 mg/kg was orally administered to monkeys at 6 h prior to DMTS testing. Each plot at 0, 4, 8 or 16 s interval were presented as the mean ± SEM within 96 trials per session (n = 3) (left). TAK-653 significantly ameliorated DMTS accuracy at 16 s delay interval (right). ^*^P ≤ 0.05 (versus vehicle group; paired *t* test). (**G**) Effect of TAK-653 on sociability deficits in the social approach-avoidance test in poly-I:C mouse. TAK-653 at 0.3 mg/kg was orally administered to mice at 1 h prior to test session. Sniffing index data were presented as the mean ± SEM (n = 7). *P ≤ 0.05 (versus control mice group; Student’s *t* test); ^#^*P* ≤ 0.05 (versus control mice group; Student’s *t* test).

The effect of TAK-653 on visual learning and memory was assessed using the novel object recognition (NOR) test in rats. TAK-653 at 0.03, 0.1 and 0.3 mg/kg, p.o. significantly improved the novelty discrimination index (NDI) (Fig. 4A). TAK-653 may enhance visual learning and memory at ≥ 0.03 mg/kg, p.o. in normal rats. In our previous study in rats, LY451646 at ≥ 1 mg/kg, p.o. improved visual learning and memory in the NOR test, although it induced seizure at 10 and 30 mg/kg, p.o.^16^. AMPA induced seizure at 30 mg/kg, i.p. and abnormal behavior such as head turning at 10 mg/kg, i.p. in rats, thus we assessed its effects on cognitive function at 3 mg/kg, i.p. and less. Surprisingly, AMPA at 0.3 to 3 mg/kg, i.p. did not improve cognitive performance in the NOR test (Fig. 4B).

The effect of TAK-653 on MK-801-induced working memory deficit in rats was evaluated using the radial arm maze (RAM) task. MK-801 (0.08 mg/kg, s.c.) disrupted the performance of well-trained rats and TAK-653 at 0.1, 0.3, 3 and 10 mg/kg, p.o. significantly ameliorated the MK-801-induced deficits (Fig. 4C). Thus, TAK-653 may enhance working memory performance over a broad dose range in hypoglutamatergic conditions. By contrast, neither LY451646 at 0.03 to 1 mg/kg, p.o. nor AMPA at 0.1 to 3 mg/kg, i.p. improve working memory in the RAM test (Fig. 4D,E).

We also characterized another AMPA-R potentiator, PF-04958242. PF-04958242 showed significant agonistic effects; PF-04958242 potently produced a Ca^2+^ increase in the absence of AMPA in rat primary hippocampal neurons (Fig. S1A). Similar to LY451646, PF-04958242 improved visual learning and memory at ≥ 0.1 mg/kg, p.o. in the NOR test, while PF-04958242 did not improve working memory in the RAM test (Fig. S1B and S1C).

The effect of TAK-653 on working memory was also assessed using the delayed match-to-sample (DMTS) paradigm in monkeys. C_max_ at 0.06 mg/kg p.o. in fasted monkeys corresponds to that at 0.1 mg/kg p.o. in rats, thus 0.06 mg/kg, p.o. of TAK-653 was used in the monkey study. TAK-653 at 0.06 mg/kg, p.o. significantly increased DMTS accuracy at a 16-s delay interval (Fig. 4F). The significant improvement in accuracy returned to the vehicle level at 48 h later, reflecting that the favorable effect was not attributable to a training effect. The beneficial effect of TAK-653 on task accuracy maintained 24 h after administration probably due to its T_max_ value (8.0 ± 0.0 h) and t_1/2_ value (9.4 ± 3.8 h) at 0.03 mg/kg, p.o. in monkeys. TAK-653 may also improve working memory in monkeys at similar plasma concentrations as observed in rats for improving visual learning, recognition memory and working memory.

We investigated the effects of TAK-653 on attention in the rat 5-choice serial reaction time task (5CSRTT). Rats with poor performance could be a useful model of ADHD^24^. Thus, sub-population analyses with a median split of the population into high and poor performing animals, were also performed based on their correct responses under vehicle treatment for each treatment block^25^. TAK-653 (0.3 mg/kg, p.o.) administration at 2 h prior to the trial showed no significant improvement in the whole population, but median split analysis (median value, 51) revealed that TAK-653 significantly increased correct responses and decreased omissions in the poor performing rats (Fig. S2). TAK-653 did not affect the premature responses. Thus, TAK-653 may enhance sustained attention in the poor performing rats.

The effect of TAK-653 on sociability deficits was evaluated using the social approach-avoidance test in the poly-I:C mouse, a developmental immune activation animal model of schizophrenia^26,27^. The sniffing index was significantly decreased in vehicle-treated poly-I:C mice compared with vehicle-treated control mice. TAK-653 at 0.3 mg/kg, p.o. significantly improved the sniffing index (Fig. 4G). TAK-653 may ameliorate abnormal social interaction.

### TAK-653 had a low risk of receptor desensitization or sensitization in vivo

**Figure 5:**
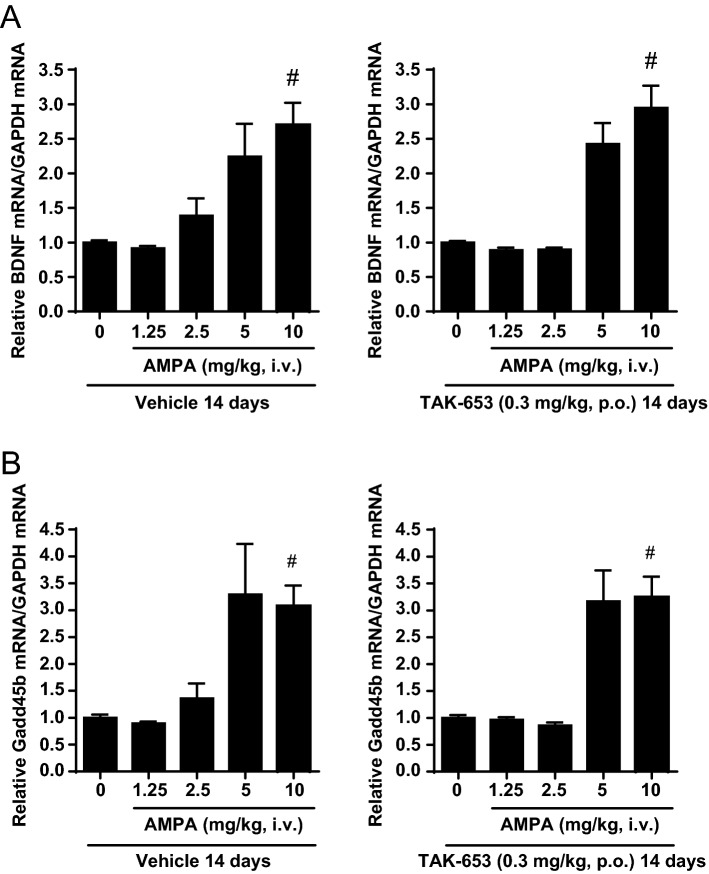
Effects of repeated treatment of TAK-653 on AMPA-induced BDNF (**A**) or Gadd45b (**B**) mRNA expression in mouse hippocampus. Vehicle or TAK-653 (0.3 mg/kg, p.o.) for 14 days were administered to mice. On the day 14, vehicle or AMPA (1.25, 2.5, 5 or 10 mg/kg, i.v.) was administered 1 h after vehicle (left) or TAK-653 (right). Tissues were isolated 3 h after AMPA administration. Data were presented as the mean ± SEM (n = 23–24). ^#^*P* ≤ 0.05 (versus vehicle-treated group; two-tailed Shirley–Williams test).

Down-regulation of AMPA-Rs or sensitization of the AMPA-R system following chronic simulation are a concern with AMPA-R activators^28,29^. TAK-653 at 0.3 mg/kg, p.o. improved social interaction in mice (Fig. 4G) and in vivo plasma exposure level at 0.1 mg/kg, p.o. were similar between rats and mice (Table S3), thus we assessed AMPA-R function after 14 days of TAK-653 administration at 0.3 mg/kg p.o. in mice. AMPA-R activation is known to induce the expression of BDNF and growth arrest and DNA-damage-inducible beta (Gadd45b) mRNA^30^. Pre-administration of TAK-653 at 0.3 mg/kg, p.o. for 14 days did not affect AMPA-induced BDNF and Gadd45b mRNA expression in the mouse hippocampus (Fig. 5A,B), suggesting a lower risk of receptor desensitization or sensitization after repetitive dosing.

### TAK-653 enhanced AMPA-R-mediated synaptic responses more potently than AMPA or LY451646 in prefrontal cortex (PFC) slices

**Figure 6:**
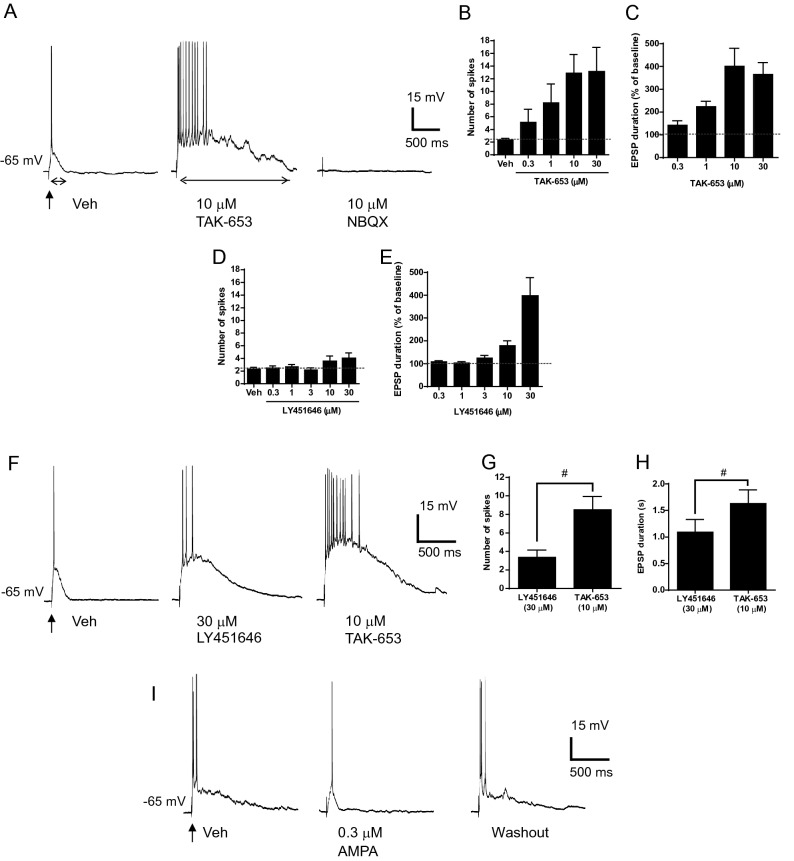
Effect of TAK-653, LY451646, and AMPA on AMPA-R-mediated EPSPs in prefrontal cortical slice. (**A**) In the presence of bicuculline (20 μM), CGP52422 (10 μM), and APV (50 μM) to block GABA_A_ and _B_ and NMDA receptors, respectively, single stimulation of glutamatergic afferents to PFC pyramidal neurons evoked polysynaptic EPSPs to induce action potential spiking. Addition of 10 μM TAK-653 for 10 min enhanced the suprathreshold response, resulting in the prolongation of action potentials trains. This response was abolished by the AMPA receptor antagonist NBQX (10 μM). (**B**, **C**) Effects of TAK-653 on the number of spikes (**B**) and duration (**C**) of AMPA-R-mediated EPSPs. Data were represented as mean ± SEM (n = 6–10). (**D**, **E**) Effects of LY451646 on the number of spikes (**D**) and duration (**E**) of AMPA-R-mediated EPSPs. (**F**) Addition of 30 μM LY451646 for 10 min enhanced the suprathreshold response. Perfusion changes from LY451646 to TAK653 caused further augmentation of spike number. (**G**, **H**) Effects of TAK-653 after removal of LY451646 on the number of spikes (**G**) and duration (**H**) of AMPA-R-mediated EPSPs. Data were represented as mean ± SEM (n = 6–9). Statistical significance between LY451646 and TAK-653 was determined by a paired *t* test with significance set at ^#^*P* ≤ 0.05. (**I**) Application of 0.3 μM AMPA for 5 min diminished the suprathreshold response (n = 6).

Desensitization of AMPA-R by agonistic effects may be a cause for reduced (or lack of) efficacy or extremely narrow bell-shaped dose-responses in cognitive improvement mediated by AMPA or LY451646; however, this hypothesis is inconsistent with their seizure risk at higher doses. To understand the underlying mechanism of action, we examined AMPA-mediated synaptic responses. In the presence of bicuculline (20 μM), CGP52422 (10 μM), and APV (50 μM) to block GABA_A_, GABA_B_ and NMDA receptors, respectively, electrical stimulation of layer I elicited the AMPA-R-mediated polysynaptic EPSPs of layer V pyramidal neurons into burst firing while the somatic membrane was maintained at approximately − 65 mV with constant current (Fig. 6A). Bath application of 0.3 to 30 μM TAK-653 for 10 min enhanced the suprathreshold polysynaptic EPSPs with a significant increase in evoked spikes (Fig. 6B) and EPSP duration (Fig. 6C) in a concentration-dependent manner. The enhancing effects of TAK-653 were maintained for at least 10 to 20 min after elimination. By blocking the synaptic responses of AMPA-R with NBQX (10 μM), the effect of TAK-653 completely disappeared (Fig. 6A). Similar to TAK-653, application of TAK-137 at 3 μM also robustly enhanced polysynaptic EPSP duration and evoked spikes (Fig. S3). Compared with TAK-653 and TAK-137, LY451646 at 0.3 to 30 μM induced moderate potentiation of suprathreshold polysynaptic EPSPs with a smaller number of spikes (Fig. 6D) and EPSP duration (Fig. 6E). Following a switch from LY451646 (30 μM) to TAK-653 (10 μM) (Fig. 6F), a clear increase in spike number (Fig. 6G) and EPSP duration (Fig. 6H) were observed, indicating no pronounced desensitization of AMPA-R by LY451646. Both TAK-653 and LY451646 did not increase the membrane potentials of recorded neurons, while AMPA (0.3 μM) increased the membrane potentials. Under these conditions, AMPA reduced polysynaptic EPSP duration and evoked spike number when the membrane was held at − 65 mV with a current injection (Fig. 6I). Thus, TAK-653 might augment the AMPA-R-mediated polysynaptic network interactions more robustly than LY451646 or AMPA.

### TAK-653 had a wider safety margin against seizures than LY451646 in rats

Cognitive improvement by TAK-137 in a variety of paradigms was observed at a similar dosage (around 0.1 mg/kg) in rats^16,31^. On the other hand, LY451646 showed dose-dependent efficacy only in the NOR test. Thus, we decided to calculate the exposure safety margins of AMPA-R potentiators based on the cognitive improvement in the NOR test and the absence of signs of seizures. TAK-653 elicited chronic and tonic convulsions in 1 animal at 4 h after dosing at 100 mg/kg, p.o. (Table S4). The exposure margins of TAK-653, calculated using the area under the plasma drug concentration–time curve (AUC_plasma_) values and plasma C_max_ values, were 1017-fold (AUC_plasma_) and 419-fold (plasma C_max_), respectively (Table 2 and S5). Exposure margins of TAK-137 and LY451646 by this protocol were 122- and 4.0-fold (AUC_plasma_), respectively, and 42- and 3.4-fold (plasma C_max_), respectively. Thus, TAK-653 may have a wider exposure margin than LY451646 in rats.

**Table 2:** Exposure margin against seizure after acute treatment in rats (plasma C_max_ and AUC_plasma_).

	TAK-653	TAK-137	LY451646
Margin based on plasma C_max_ (fold)	419	42	3.4
Margin based on AUC_plasma_ (fold)	1017	122	4.0

## Discussion

In neuropsychiatric disorders, functional enhancement of AMPA-R has the potential to improve cognitive deficits in several cognitive domains including executive function, attention, and working memory^32^. Enhancement of AMPA-R-mediated neurotransmission may also lead to rapid antidepressant action^9,33–35^. Thus, AMPA-R potentiators could be promising therapeutic drugs for multiple CNS disorders. However, seizure liability and narrow bell-shaped dose-responses might have restricted the development of AMPA-R potentiators as therapeutic drugs.

We hypothesized that the agonistic effects of some AMPA-R potentiators are associated with their seizure risks and bell-shaped dose-response effects. In fact, TAK-137, an AMPA-R potentiator with lower agonistic effects than LY451646, showed lower risks of seizure and bell-shaped dose-response^16^. In this study, we asked whether an AMPA-R potentiator with lower agonistic effects than TAK-137 (i.e. an AMPA-R potentiator with virtually no agonistic effect) may have lower seizure risks or if the beneficial effect is lost. To answer this question, we discovered a novel AMPA-R potentiator TAK-653 with extremely lower agonistic effects. [^3^H]-TAK-653 bound to the intradimer interface formed by the ligand-binding core of AMPA-R subunits in a glutamate-dependent manner. This intradimer interface is known to undergo conformational changes upon glutamate binding^36,37^. Co-crystallization studies suggested that TAK-653 exhibited glutamate-dependent structural interference at Ser750, located in the intradimer interface of GluA2o with larger steric repulsion in the absence of glutamate. In fact, the introduction of a mutation to Ser743 (S743A) in GluA1i (corresponding to Ser750 in GluA2o) to lower the steric interaction between AMPA-R and TAK-653 reduced the glutamate threshold for AMPA-R activation by TAK-653. Thus, like TAK-137, steric interaction at Ser743 in the channel-closed state may be related to the lower agonistic effects of TAK-653^16^. Both TAK-137 and TAK-653 had similar potential in the augmentation of agonist-elicited AMPA-R function, while TAK-653 had a lower agonistic effect than TAK-137 on both Ca^2+^ influx and whole-cell currents using primary neurons.

Quite interestingly, like TAK-137, TAK-653 produced a potent cognitive improvement in a wide range of cognitive domains. Neither LY451646 nor AMPA, however, improved working memory in the RAM test and AMPA did not improve recognition memory in the NOR test in rats. The effects of LY451646 in the RAM test were evaluated at around 1 mg/kg, p.o., a dose at which LY451646 produced efficacy in the NOR test. Therefore, brain exposure of LY451646 under the conditions for the RAM test was high enough to explore its therapeutic potential for working memory. The effects of AMPA were investigated at a broader range of doses from 0.3 to 3 mg/kg, i.p. because AMPA produced abnormal behavior at 10 mg/kg, i.p. Note that all three compounds induced convulsions at higher exposure, thus AMPA-R activation by all of them was clear. Therefore, facilitation of physiologically active AMPA-Rs, but not increasing the number of active AMPA-Rs by stimulating resting receptors, is key to producing potent cognitive improvements through AMPA-R activation. In line with this hypothesis, PF-04958242, with prominent agonistic activity for AMPA-R on rat primary hippocampal neurons, improved visual learning and memory but it did not improve working memory.

In isolated primary neurons, AMPA dose-dependently increased intracellular Ca^2+^ levels and AMPA-R-mediated currents (Fig. 2). Under these conditions, TAK-653 and LY451646 showed similar activity in the potentiation of AMPA-elicited Ca^2+^ increases and TAK-653 was 2.3 times less potent than LY451646 in the potentiation of AMPA-elicited currents (Table 1 and fig. S4). To our surprise, TAK-653 produced more robust potentiation of the AMPA-R component of the synaptic responses, especially the spike number, compared with LY451646 in the PFC neuronal network (Fig. 6). TAK-653 could enhance polysynaptic EPSPs after LY451646 exposure, thus it is unlikely that LY451646 reduced the number of AMPA-Rs by rapid desensitization during the 10 min for slice preparation. Furthermore, AMPA (0.3 μM) reduced polysynaptic EPSP duration and evoked spikes. Most neurons transform thousands of synaptic inputs into specific patterns of action potential output^38,39^. Nonspecific stimulation of resting AMPA-R by agonistic effects could activate multiple neural circuits simultaneously. The resulting continuous and repetitive synaptic inputs on single neurons connected through their multiple pathways may cause sublinear summation, such as through the activation of postsynaptic conductance^40,41^, with the reduced signal-to-noise ratio of an evoked response. TAK-653 might remarkably increase firing rate via strictly regulated agonist-dependent activation of AMPA-R, which may lead to potent cognitive improvement (Table S7). In line with this observation, similar to TAK-653, TAK-137, which improves cognitive functions in multiple paradigms, also robustly potentiated the AMPA-R component of the synaptic responses.

As previously reported, TAK-137 has little agonistic activity. However, compared with TAK-137, TAK-653 may have even lower agonistic activity. TAK-653 has a wider exposure margin than TAK-137 between cognitive improvement and seizure in rats, thus, this small difference in the agonistic effects of TAK-653 and TAK-137 in vitro likely related to their seizure susceptibility at higher dose ranges. Besides seizure liability, a bell-shaped dose-response might have limited the development of AMPA-R potentiators as drugs^42,43^. Given the heterogeneity of human metabolic profiles that may be associated with larger variations in pharmacokinetic profiles of drugs, bell-shaped dose-responses may be a significant disadvantage even with deliberate design of dose selection^44^. TAK-653 may overcome these issues because it showed cognitive improvements at a wider dose range and had a low risk of receptor desensitization or sensitization after 14 days of repetitive dosing.

Multiple findings have suggested that ketamine can produce antidepressant activity through a rapid disinhibition of pyramidal neurons and subsequent glutamate burst, followed by postsynaptic AMPA-R activation^45^. Based on the enhancement of AMPA-R-mediated EPSPs, TAK-653 could robustly enhance the activity of postsynaptic AMPA-Rs. These data further support the potential antidepressant action of TAK-653, although detailed preclinical studies to assess antidepressant effects of TAK-653 are needed.

In summary, we found that agonistic effects may significantly impair the function of AMPA-R potentiators in synaptic transmission and interfere with cognitive improvement. In fact, TAK-653, an AMPA-R potentiator with minimal agonistic activity, substantially enhanced synaptic AMPA-R responses and potently improved cognitive functions in multiple tasks at a wider dose range and with a broader safety margin against seizure. TAK-653 could be effective for multiple CNS diseases with cognitive dysfunction, and depression. TAK-653 is currently being developed for the treatment of depressive disorders.

## Materials and methods

The care and use of the animals and the experimental protocols used in this research were approved by the Experimental Animal Care and Use Committee of Takeda Pharmaceutical Company Limited and conducted in accordance with the guidelines. The animal care and use program is accredited by the American Association for Accreditation of Laboratory Animal Care (AAALAC) International’s Council on Accreditation. The AAALAC sets standards that call for the humane care and use of laboratory animals by enhancing animal well-being, improving the quality of research and advancing scientific knowledge relevant to humans and animals. All experiments were carried out in compliance with the ARRIVE guidelines.

### Animals

ICR and C57BL/6 J mice were supplied by CLEA Japan Inc. (Tokyo, Japan). Sprague–Dawley rats were supplied by Charles River Laboratories Japan, Inc. (Yokohama, Japan). Long-Evans rats were purchased from Japan SLC Inc. (Hamamatsu, Japan). Male cynomolgus monkeys (Macaca fascicularis) were supplied by Keari Co., Limited (Osaka, Japan). The animals were housed in a light-controlled room (12-h light/dark cycle, with lights on at 7:00 AM) and were habituated more than 1 week prior to experiments.

### Chemicals

TAK-653 9-[4-(Cyclohexyloxy)phenyl]-7-methyl-3,4-dihydropyrazino[2,1-c][1,2,4]thiadiazine 2,2-dioxide, LY451646 and [^3^H]-TAK-653 were synthesized by Takeda Pharmaceutical Company Limited. [^3^H]-HBT1 was synthesized by Sekisui Medical Company Limited. S-AMPA, cyclothiazide, CGP52422, APV and bicuculline methochloride were obtained from Tocris Bioscience (Bristol, UK). L-glutamate and NBQX were obtained from WAKO Pure Chemicals (Tokyo, Japan). ( +)-MK-801 hydrogen maleate was obtained from Sigma-Aldrich (St Louis, MO). For in vivo studies, TAK-653 was suspended in 0.5% (w/v) methylcellulose in distilled water and orally administered.

### SPA binding assay

The SPA assays were performed as previously described^21^, with some modifications. First, 62.5 μg YSi (2–5 μm) copper his-tag SPA beads (PerkinElmer Inc., Waltham, MA) and 0.25 μg His-tagged GluA2o LBD protein (His-LBD) were incubated in 100 μL PBS containing 0.01% NP-40 and 100 μM glutamate for the [^3^H]-HBT1/LBD binding assay, containing 0.01% NP-40 and the indicated amount of glutamate for the [^3^H]-TAK-653/LBD binding assay or containing 0.001% Triton X-100 for the [^3^H]-AMPA/LBD binding assay in 96-well Luminunc plates (Thermo Fisher Scientific Inc.) overnight at 4 °C. Subsequently, test compound and tritium-labeled ligand (40 nM [^3^H]-HBT1, 20 nM [^3^H]-TAK-653 or 20 nM [^3^H]-AMPA) were added to each well. Specific binding was defined as total binding minus nonspecific binding, which was estimated in the presence of 0.25 μg of control protein (His-tagged macrophage MIF protein) instead of His-LBD or in the presence of 3 mM AMPA. Radioactivity derived from the bound radio ligand was measured using a microplate scintillation counter (TopCount NXT, PerkinElmer Inc.).

### X-ray crystallography of the GluA2o LBD/compound complex

The human GluA2o LBD was prepared as described previously^21^. Methods are described in the Supplementary Information.

### Ca^2+^ influx assay using cell lines expressing AMPA-Rs

Ca^2+^ influx assays using human GluA1/flip and human stargazin co-expressing CHO cells plated at 3 × 10^4^ cells/well in 96-well Black Clear plates (Corning Incorporated, Corning, NY) were performed as previously described^21^, with some modifications. For evaluation of the species difference, rat GluA1/flip expressing CHO cells were plated. For mutant channels, the human GluA1i S743A mutant was generated using the In-Fusion HD Cloning Kit (Takara Bio Inc., Kusatsu, Japan) according to the manufacturer's protocol and transiently introduced to CHO cells by Gene Pulser II Electroporation System (Bio-Rad Laboratories, Inc., Hercules, CA). Relative increases of intracellular Ca^2+^ levels were monitored for 3 min with a fluorometric imaging plate reader (CellLux, Perkin Elmer Life and Analytical Sciences, Inc., Shelton, CT). Details are described in the Supplementary Information.

### Ca^2+^ influx assay using primary neurons

Ca^2+^ influx assay using primary neurons was performed as previously described^21^. After 5 days of culture, the cells plated on poly-D-lysine coated 96-well plates (Corning Incorporated) at 2 × 10^4^ or 5 × 10^4^ cells/well were used for experiments with PF-04958242 or TAK-653, respectively. Fluorescent calcium indicator dye solution (Calcium4 assay Kit, Dojindo, Kumamoto, Japan) in Ca^2+^ reaction buffer (DMEM, HEPES and BSA) containing Probenecid (Dojindo) was added and incubated for 60 min in 5% CO_2_ at 37 °C. After washing once, the relative increases in intracellular Ca^2+^ levels stimulated by compounds in the presence and absence of AMPA were monitored. Details are described in the Supplementary Information.

### Whole-cell patch clamp recording using primary neurons

Whole-cell patch clamp recording using primary neurons between 11 and 20 days in vitro was performed as previously described^21^. Patch electrodes with tip resistances ranging from 3 to 5 MΩ were filled with an intracellular solution containing: 135 mM CsCl, 1 mM MgCl_2_, 10 mM HEPES, 10 mM EGTA, 4 mM MgATP, and 0.3 mM Na_2_GTP, adjusted to pH7.3 with CsOH (osmolality 275–295 mosm/L). The extracellular solution contained: 140 mM NaCl, 4 mM KCl, 2 mM CaCl_2_, 1 mM MgCl_2_, 10 mM HEPES, 5 mM NaHCO_3_, 10 mM D( +)-glucose, and 0.001 mM TTX, adjusted to pH7.4 with NaOH (osmolality 300–315 mosm/L). All experiments were carried out at room temperature. Neurons were voltage-clamped at − 80 mV. Steady-state inward currents were evoked by the application of AMPA and AMPA potentiator via Y-tube perfusion system. Signals were recorded using a Multiclamp 700B amplifier (Molecular Devices, LLC, Orleans Drive Sunnyvale, CA), digitized using a Digidata 1440A interface board, filtered between 2 kHz, sampled at 10 kHz, and analyzed with pClamp10 software. Details are described in the Supplementary Information.

### BDNF production in primary neurons

BDNF production in primary neurons was measured as previously described^21^. Primary cultures of hippocampal neuronal cells at a density of 5 × 10^4^ cells/well plated on poly-L-lysine coated 96-well plates (Sumitomo Bakelite) were treated with compounds in the presence or absence of AMPA (1 μM) and cultured in a humidified CO_2_ incubator with 5% CO_2_ at 37 °C for 24 h. The cells were washed once using phosphate buffered saline and were collected using 60 μL of lysis buffer (20 mM Tris-HCl at pH 8.0, 137 mM NaCl, 10% glycerol, 1% NP-40, 1% protease inhibitor cocktail [Sigma-Aldrich, St. Louis, MO]). Concentrations of BDNF protein were measured using BDNF ELISA Kits (Promega, Fitchburg, WI).

### Whole-cell patch clamp recording in acute brain slices

Coronal PFC slices were prepared from male Sprague–Dawley rats (10- to 14-day-old). The slices including layer V PFC pyramidal neurons were perfused at a flow rate of 1–2 ml/min with recording aCSF containing the following: 124 mM NaCl, 5 mM KCl, 1.2 mM NaH_2_PO_4_, 1.5 mM MgCl_2_, 2.5 mM CaCl_2_, 10 mM glucose, 24 mM NaHCO_3_ bubbled with carbogen (95% O_2_; 5% CO_2_). Current clamp recordings at holding potential of approximately − 65 mV with constant current were carried out at 32–33 °C using a pipette filled with an intracellular solution consisting of: 140 mM K-gluconate, 4 mM KCl, 10 mM HEPES, 0.2 mM EGTA, 4 mM MgATP, 0.3 mM Na_2_GTP, pH 7.3 with KOH in the presence of bicuculline (20 μM), CGP52422 (10 μM), and APV (50 μM) to block GABA_A_ and _B_ and NMDA receptors, respectively. Methods are described in the Supplementary Information.

### mRNA expression measurement in vivo

Male ICR mice at 7 weeks old were treated with orally administered vehicle or TAK-653. For the repeated dose study, TAK-653 (0.3 mg/kg, p.o.) or vehicle was administered for 14 days. AMPA at designated doses was intravenously (i.v.) administered 1 h after oral administration. Three hours after i.v. administration of AMPA, right hippocampi were isolated from the mice by euthanasia and acutely frozen on dry ice. Total RNA from mouse hippocampus was extracted with RNeasy 96 kit (Qiagen, Hilden, Germany). Reverse transcription of total RNA was performed using the High-capacity cDNA Archive kit (Life Technologies, Carlsbad, CA). Real-time quantitative PCR was carried out using an ABI PRISM 7900HT sequence detection system (Life Technologies) using PCR reagents (Nippon Gene Co. Limited, Toyama, Japan). Primers used for BDNF analyses were as follows: BDNF forward primer, 5’-ACCATAAGGACGCGGACTTGT-3’; BDNF reverse primer, 5’-GGAGGCTCCAAAGGCACTTGA-3’; BDNF TaqMan probe, 5’-FAM- ACACTTCCCGGGTGATGCTCAGCA-TAMRA-3’. Primers used for Gadd45b analyses were as follows: Gadd45b forward primer, 5’-CACCCTGATCCAGTCGTTCTG-3’; Gadd45b reverse primer, 5’-GCGCCAGCCTCTGCAT-3’; Gadd45b TaqMan probe, 5’'-FAM-CAATGACATTGACATCGTCCGGGTATCAG-MGB-3’. Primers used for Glyceraldehyde-3-phosphate dehydrogenase (GAPDH) analysis were as follows: GAPDH forward primer, 5’-TGAGCAAGAGAGGCCCTATCC-3'; GAPDH reverse primer, 5'-CCCTCCTGTTATTATGGGGGTCT-3'; GAPDH TaqMan probe, 5'-FAM-CCCCAACACTGAGCATCTCCCTCACAA-TAMRA-3'. Concentrations of GAPDH mRNA were used for internal controls and relative BDNF or Gadd45b mRNA/GAPDH mRNA levels were calculated.

### Novel object recognition test

NOR tests were performed using male Long-Evans rats at 6 weeks old as previously described^46^, with some modifications. On day 1, rats were allowed to habituate to the empty test box (a gray-colored polyvinyl chloride box (40 × 40 × 50 cm)) for 10 min individually. Testing comprised two 3-min trials called the acquisition trial and the retention trial that were separated by 48 h inter-trial intervals (ITIs). On day 2, in the acquisition trial, rats were allowed to explore two identical objects (A1 and A2) for 3 min. On day 4, in the retention trial, rats were again allowed to explore a familiar object (A3) and a novel object (B) for 3 min. The object exploration was defined as rats' licking, sniffing or touching the object with forelimbs while sniffing. TAK-653 or AMPA was orally or intraperitoneally, respectively, 2 h or 0.5 h prior to the acquisition and the retention trials. The NDI was calculated using the following equation: novel object interaction/total interaction × 100 (%).

### Radial arm maze test

RAM tests were performed using 9-week-old male Long-Evans rats as previously described with a minor modification^31^. Each arm was 50 cm long, 10 cm wide and 40 cm high, and the maze was elevated 50 cm above the floor. Long-Evans rats were food-restricted to 85% of free-feeding body weight throughout the experimental period. Rats were well trained to collect pellets placed on the edge of each arm. The learning criterion for the testing session was defined as 2 errors or fewer for 2 consecutive days. In the testing session, each rat was placed on the maze facing the fixed arm at the start of the trial. The entry of rats into each arm was recorded in sequence until all pellets in the 8 arms were consumed, or 5 min had elapsed. TAK-653, LY451646 or AMPA was administered 1.5 h, 1 h or 0 h prior to the administration of vehicle or MK-801, respectively. Thirty min after dosing of vehicle or MK-801, rats were placed on the maze. Details are described in the Supplementary Information.

### Delayed match-to-sample tasks

DMTS tasks were performed using 4–6 years old male cynomolgus monkeys (Macaca fascicularis) weighing 4–6 kg as previously described^16^, using a Cambridge Neuropsychological Test Automated Battery (CANTAB) system (CeNes, Cambridge, UK). Monkeys were maintained at 80% of free-feeding body weight throughout the experiment. Details are described in the Supplementary Information.

### Maternal immune activation induction

Poly-I:C (5 mg/kg) dissolved in sterile pyrogen-free 0.9% NaCl (control) solution was administered to pregnant female C57BL/6J mice on gestation day 15 (GD15) via the intravenous route at the tail vein with a volume of 5 ml/kg.

### Social approach and avoidance test

The apparatus was a three-chamber gray acryl box (53 × 19 × 21.5 cm, outer chamber: 19.3 × 19 × 21.5 cm), and dividing walls were made from clear acryl plates with gates. In the two outer chambers, transparent acryl cylinders with small holes (cylinder: 8.2 cm φ × 20.5 cm, hole: 1.3 cm φ) were placed to avoid direct physical interaction between a target animal and a test animal. Target animals were C57BL/6 J mice of the same age as test animals and no previous contact with them. On the test day, TAK-653 (0.3 mg/kg, p.o.) or vehicle was administered 1 h before the testing session. Each test animal was introduced in the middle chamber of test apparatus for 3 min with the gates being closed by partitions. Then the partitions were removed gently for the test animal to explore all three chambers freely for 5 min. Sniffing index was calculated as the index of sociability using the following equation:

Sniffing index = (Sniffing time to cylinder containing target mouse (s) − Sniffing time to empty cylinder (s))/ (Total sniffing time (s)).

### Evaluation of convulsion

The data were extracted from the studies conducted in accordance with the Good Laboratory Practice Regulation. Male Sprague–Dawley rats obtained at 4 weeks old showed no observable abnormalities in clinical signs during the predose period. The dose levels in the first study and second study were set at "100, 15 and 5 mg/kg, p.o." and "50, 15 and 5 mg/kg, p.o.", respectively. The dosing suspensions were administered into the stomach via a catheter in the morning at the dose volume of 5 mL/kg. Cage side observations to confirm tonic or chronic convulsions were conducted before and at 1, 2, 4, 8 and 24 h after dosing in the first study and before and at 1, 4 and 24 h after dosing in the second study.

### Measurement of plasma concentration of compounds

The Sprague–Dawley rats treated with orally administered TAK-653 were decapitated at each time point, and trunk blood was collected into 1.5-mL centrifuge tubes. Plasma was separated from the blood samples by centrifugation. The concentrations of TAK-653 in the plasma were determined using liquid chromatography/tandem mass spectrometry (LC/MS/MS).

### Statistics

Student's *t* tests (for homogeneous data) or Aspin-Welch's tests (for nonhomogeneous data) were carried out to assess the statistical significance of differences between the 2 groups. In experiments with multiple doses of test compounds, statistical significance was analyzed using the two-tailed Williams’ test (for homogeneous data) or the two-tailed Shirley-Williams test (for nonhomogeneous data). In DMTS experiments, we compared the performance at the 16-s delay interval by paired *t* test (vs. vehicle group). *P* values ≤ 0.05 were considered significant.

## Supplementary Information

Supplementary Information.
