# Author Correction: Strictly regulated agonist-dependent activation of AMPA-R is the key characteristic of TAK-653 for robust synaptic responses and cognitive improvement

**DOI:** 10.1038/s41598-021-94772-7

**Published:** 2021-07-21

**Authors:** Atsushi Suzuki, Akiyoshi Kunugi, Yasukazu Tajima, Noriko Suzuki, Motohisa Suzuki, Masashi Toyofuku, Haruhiko Kuno, Satoshi Sogabe, Yohei Kosugi, Yasuyuki Awasaki, Tomohiro Kaku, Haruhide Kimura

**Affiliations:** 1 Neuroscience Drug Discovery Unit, Research, Takeda Pharmaceutical Company Limited, 26-1, Muraoka-Higashi 2-chome, Kanagawa, Fujisawa 251-8555 Japan; 2 Bio-Molecular Research Laboratories, Research, Takeda Pharmaceutical Company Limited, Fujisawa, Japan; 3 Drug Metabolism and Pharmacokinetics Research Laboratories, Research, Takeda Pharmaceutical Company Limited, Fujisawa, Japan; 4 Drug Safety Research and Evaluation, Research, Takeda Pharmaceutical Company Limited, Fujisawa, Japan

Correction to: *Scientific Reports* 10.1038/s41598-021-93888-0, published online 15 July 2021

The original version of this Article contained an error in Figures 3 and 5 where the y-axis did not display correctly in panel B. The original Figures 3 and 5 and their accompanying legends appear below.

**Figure 3:**
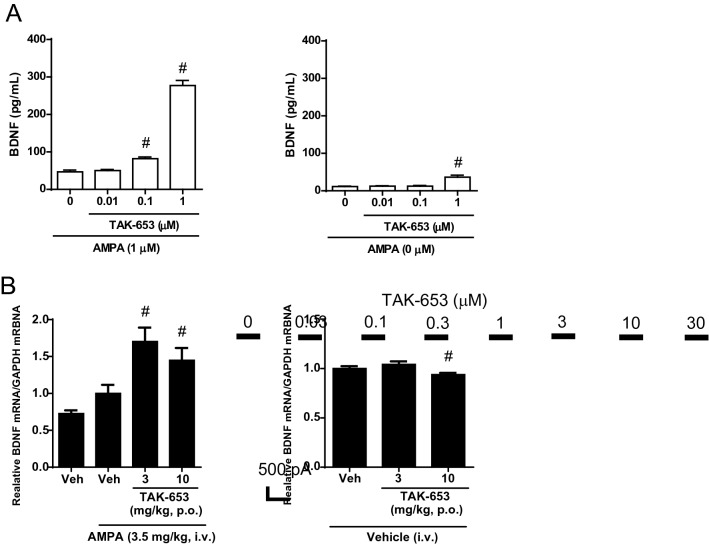
Effects of TAK-653 on BDNF expression in vitro and in vivo. (**A**) Effects of TAK-653 on BDNF protein levels in rat primary hippocampal neurons. Cells were treated with AMPA (0 or 1 μM) and TAK-653 (0.01, 0.1, 1 μM) for 24 h and then were collected using lysis buffer. Cells in control group were treated with 1 μM AMPA and DMSO. Values were expressed as pg per mL. Data are represented as mean ± SD (n = 3). Statistical significance was determined by a two-tailed Williams’ test with significance set at ^#^*P* ≤ 0.05 (versus control group; two-tailed Williams’ test). (**B**) Effects of TAK-653 on BDNF mRNA in hippocampus in AMPA (3.5 mg/kg, i.v.)-treated mice. TAK-653 (3 and 10 mg/kg, p.o.) was administered to mice 1 h before the administration of AMPA (3.5 mg/kg, i.v.) (left) or vehicle (right). Tissues were isolated 3 h after AMPA administration. Data were presented as the mean ± SEM (n = 23–24). ^#^*P* ≤ 0.05 (versus vehicle-treated group; two-tailed Williams’ test).

**Figure 5:**
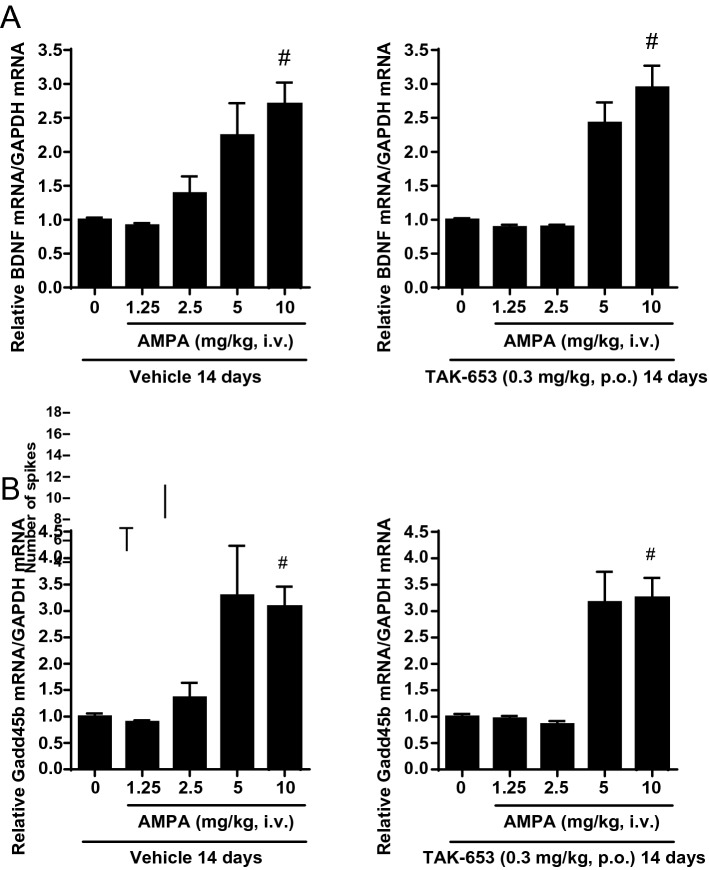
Effects of repeated treatment of TAK-653 on AMPA-induced BDNF (**A**) or Gadd45b (**B**) mRNA expression in mouse hippocampus. Vehicle or TAK-653 (0.3 mg/kg, p.o.) for 14 days were administered to mice. On the day 14, vehicle or AMPA (1.25, 2.5, 5 or 10 mg/kg, i.v.) was administered 1 h after vehicle (left) or TAK-653 (right). Tissues were isolated 3 h after AMPA administration. Data were presented as the mean ± SEM (n = 23–24). ^#^*P* ≤ 0.05 (versus vehicle-treated group; two-tailed Shirley–Williams test).

The original Article has been corrected.

