# Supplementary material for: Strictly regulated agonist-dependent activation of AMPA-R is the key characteristic of TAK-653 for robust synaptic responses and cognitive improvement
Source: Sci Rep. 2021 Jul 15;11:14532. doi: 10.1038/s41598-021-93888-0 (PMC8282797; doi:10.1038/s41598-021-93888-0)
Supplement: Supplementary file 1 — Supplementary Information. [file 41598_2021_93888_MOESM1_ESM.docx]

**Supplementary Information**

**Strictly regulated agonist-dependent activation of AMPA-R is the key characteristic of TAK-653 for robust synaptic responses and cognitive improvement**

Atsushi Suzuki ^1^, Akiyoshi Kunugi ^1^, Yasukazu Tajima ^1^, Noriko Suzuki ^1^, Motohisa Suzuki ^1^, Masashi Toyofuku ^1^, Haruhiko Kuno ^2^, Satoshi Sogabe ^2^, Yohei Kosugi ^3^, Yasuyuki Awasaki ^4^, Tomohiro Kaku ^1^, and Haruhide Kimura ^*,1^

^1^Neuroscience Drug Discovery Unit, Research, Takeda Pharmaceutical Company Limited, Fujisawa, Japan; ^2^Bio-Molecular Research Laboratories, Research, Takeda Pharmaceutical Company Limited, Fujisawa, Japan; ^3^Drug Metabolism and Pharmacokinetics Research Laboratories, Research, Takeda Pharmaceutical Company Limited, Fujisawa, Japan; ^4^Drug Safety Research and Evaluation, Research, Takeda Pharmaceutical Company Limited, Fujisawa, Japan

**^*^Address correspondence to:**

Dr. Haruhide Kimura, Neuroscience Drug Discovery Unit, Research, Takeda Pharmaceutical Company Limited, 26-1, Muraoka-higashi 2-chome, Fujisawa, Kanagawa 251-8555, Japan.

Phone number: (+81) 466321859

E-mail: haruhide.kimura@takeda.com

**Supplementary Materials and Methods**

**Crystallography of the GluA2o LBD/compound complex**

The human GluA2o-LBD was prepared as described previously ([Kunugi *et al*, 2018](#_ENREF_3)). The complex was prepared by incubation of GluA2o-LBD with a 3-fold molar excess of the compound on ice for a few h prior to crystallization experiments. Crystals were grown at 4°C using the vapor diffusion method in sitting-drop technique, and the drops contained a 1:1 (v/v) ratio of protein solution to reservoir solution. The reservoir solution contained 11-18% PEG 3350, 0.1 M sodium acetate, 0.1 M zinc acetate, pH 4.3-5.4.　Prior to data collection, crystals were immersed in mother liquor solution containing 30% glycerol and flash frozen in liquid nitrogen. Diffraction data were collected from a single crystal at Advance Light Source on beam line 5.0.3 using a Quantum210 CCD detector (ADSC) under a 100 K nitrogen cryostream. Data sets were reduced and scaled using HKL2000 ([Otwinowski and Minor, 1997](#_ENREF_5)). Structures were solved by molecular replacement with MOLREP ([Vagin and Teplyakov, 2010](#_ENREF_7)) from the CCP4 suites ([Winn *et al*, 2011](#_ENREF_8)) using the coordinates of the GluA2-LBD associated with L-glutamate (PDB code 1FTJ) as a search model. The structures were refined through an iterative procedure utilizing REFMAC ([Murshudov *et al*, 2011](#_ENREF_4)) followed by model building in COOT ([Emsley *et al*, 2010](#_ENREF_2)). The dictionary files for the ligands were prepared using AFITT (OpenEye Scientific Software, Cambridge, MA). The final models were validated using Molprobity ([Chen *et al*, 2010](#_ENREF_1)). Crystallographic processing and refinement statistics are summarized in table S6. All structural figures were generated using PyMOL (Schrödinger, LLC, New York, NY).

**Ca^2+^ influx assay using cell lines expressing AMPA receptors**

Human GluA1/flip and human stargazin co-expressing CHO cells were plated at 3×10^4^ cells/well in 96-well Black Clear plate (Corning Incorporated, Corning, NY). For subunit selectivity study, human AMPA-Rs (GluA1-4i, GluA1-4o and GluA1i mutants) and human TARPs γ2 co-expressing CHO cells were plated. For evaluation of the species difference, rat GluA1/flip expressing CHO cells were plated. For mutant channels, Human GluA1i S743A mutant was generated by In-Fusion HD Cloning Kit (Takara Bio Inc., Kusatsu, Japan) according to manufacturer's protocol. Each hGluA1i mutant vector was transiently introduced to CHO cells by Gene Pulser II Electroporation System (Bio-Rad Laboratories, Inc., Hercules, CA) and incubated for 6 h in 5% CO_2_ at 37°C. Then, cells were harvested and plated. These cells were seeded at 3×10^4^ cells/well in 96-well Black Clear plate and incubated for 24 h in 5% CO_2_ at 37°C. After removal of culture medium, 100 µL of fluorescent calcium indicator dye solution (Calcium5 assay Kit, Molecular Devices, Inc., Sunnyvale, CA) in Ca^2+^ reaction buffer (Hanks' Balanced Salt Solutions, 10 mM HEPES and 0.1% BSA) containing 1.25 mM Probenecid (Lifetechnologies Corporation) was added and incubated for 60 min in 5% CO_2_ at 37°C. After 60 min incubation, cells were washed once by 100 µL of Ca^2+^ reaction buffer. 100 µL of fluorescent calcium indicator dye solution was added to each well again. Relative increases of intracellular Ca^2+^ levels by compounds in the presence and absence of 3 mM L-glutamate were monitored for 3 min by fluorometric imaging plate reader (CellLux, Perkin Elmer Life and Analytical Sciences, Inc., Shelton, CT). Activity of compound was defined as integration value of fluorescence value of each detection point. For experiments with 3 mM L-glutamate, 0% was defined as activity in the presence of 3 mM L-glutamate and DMSO, while 100% was defined as activity in the presence of 3 mM L-glutamate and 300 µM Cyclothiazide. For L-Glutamate free experiments, 0% was defined as activity in the presence of only DMSO, while 100% was defined as activity in the presence of 3 mM L-glutamate and 300 µM cyclothiazide or 10 µM LY451646. EC_50_ values were calculated by logistic regression analysis.

**Preparation of rat primary neurons**

Primary cultures of hippocampal neuronal cells were prepared from fetal Sprague-Dawley rats at 19 days of gestation. Rat embryos were decapitated and their whole brains were isolated. Hippocampi were dissected in ice-cold Hanks' Balanced Salt Solutions (HBSS) under a microscope and then dispersed into cells using a neural cell dispersion kit (Sumitomo Bakelite Co., Ltd., Tokyo, Japan). The dissociated cells were suspended in Neurobasal medium containing B27 supplement (Thermo Fisher Scientific Inc.), 2 mM L-glutamine (Lonza, Basel, Switzerland), 100 U/mL penicillin (Lonza), 100 μg/mL streptomycin (Lonza) and 20 μg/mL gentamicin sulfate (Lonza). The cells were plated on poly-D-lysine coated 96-well plates (Corning Incorporated) for Ca^2+^ influx assay or on poly-L-lysine coated 96-well plates (Sumitomo Bakelite) for measurement of BDNF protein levels and cultured in a humidified CO_2_ incubator with 5% CO_2_ at 37°C.

**Ca^2+^ influx assay using primary neurons**

After 5 days of culture, the cells at 2×10^4^ or 5×10^4^ cells/well were used for experiments with PF-04958242 or TAK-653, respectively. For TAK-653, after removal of culture medium, 75 µL of fluorescent calcium indicator dye solution (Calcium4 assay Kit, Dojindo, Kumamoto, Japan) in Ca^2+^ reaction buffer (DMEM, 10 mM HEPES and 0.1% BSA) containing 1.25 mM Probenecid (Dojindo) was added and incubated for 60 min in 5% CO_2_ at 37°C and washed once using 75 µL of fluorescent calcium indicator dye solution. After addition of 75 µL of fluorescent calcium indicator dye solution to each well again, the relative increases in intracellular Ca^2+^ levels by compounds in the presence and absence of 5 μM AMPA were monitored for 8.5 min by fluorometric imaging plate reader. Activity of the compound was defined as fluorescence intensity integrated over the entire period of measurement. Intensity of 0% was defined as activity in the presence of only DMSO, while 100% was defined as activity in the presence of 5 μM AMPA and 10 μM HBT1. For PF-04958242, after removal of culture medium, 90 µL of fluorescent calcium indicator dye solution (Calcium4 assay Kit, Dojindo) in Ca^2+^ reaction buffer (DMEM, 20 mM HEPES and 0.1% BSA) containing 2.5 mM Probenecid (Dojindo) was added and incubated for 60 min in 5% CO_2_ at 37°C. After addition of 90 µL of fluorescent calcium indicator dye solution to each well again, the relative increases in intracellular Ca^2+^ levels by compounds in the presence and absence of 1 μM AMPA were monitored for 2 min in a microplate reader (CellLux, PerkinElmer, Waltham, MA, USA). Activity of the compound was defined as fluorescence intensity integrated over the entire period of measurement. 100% was defined as activity in the presence of 1 μM AMPA and 10 μM HBT1, while intensity of 0% was defined as activity in the presence of 1 μM AMPA or only DMSO for potentiator effect or agonistic effect, respectively. EC_50_ values were calculated by logistic regression analysis.

**Whole-cell patch clamp recording in primary neurons**

Whole-cell patch clamp technique was used for recording current from cultured rat hippocampal neurons between 11-20 days in vitro. Patch electrodes with tip resistances ranging from 3 to 5 MΩ were filled with an intracellular solution containing: 135 mM CsCl, 1 mM MgCl_2_, 10 mM HEPES, 10 mM EGTA, 4 mM Mg-ATP, 0.3 mM Na_2_-GTP, adjusted to pH7.3 with CsOH (osmolality 275-295 mosm/L). The extracellular solution contained: 140 mM NaCl, 4 mM KCl, 2 mM CaCl_2_, 1 mM MgCl_2_, 10 mM HEPES, 5 mM NaHCO_3_, 10 mM D(+)-glucose, and 0.001 mM TTX, adjusted to pH7.4 with NaOH (osmolality 300-315 mosm/L). All experiments were carried out at room temperature and performed using an Axopatch-1D amplifier with pCLAMP 9 software (Molecular Devices, LLC, Orleans Drive Sunnyvale, CA), low-pass filtered at 2 kHz, and stored on the computer hard-disk for off-line analysis. Neurons were voltage-clamped at –80mV. Steady-state inward currents were evoked by the application of AMPA and AMPA potentiator via Y-tube perfusion system. AMPA-R potentiator was applied 20 s prior to 10-s AMPA stimulus. The current at all drug concentrations tested was normalized by the steady-state current induced by 1 μM AMPA. In the absence of AMPA, the maximum current during 60-s application of AMPA-R potentiator was normalized by the steady-state current induced by 100 μM AMPA.

**Whole-cell patch clamp recording in acute brain slices**

Coronal prefrontal cortex (PFC) slices were prepared from male Sprague-Dawley rats (10- to 14-day-old). Animals were sacrificed following acute decapitation, and their brain was quickly removed and placed in an ice-cold modified artificial cerebrospinal fluid (aCSF) of the following composition: 92 mM N-methyl-D-glucamine (NMDG), 2.5 mM KCl, 1.25 mM NaH_2_PO_4_, 30 mM NaHCO_3_, 20 mM HEPES, 25 mM glucose, 2 mM thiourea, 5 mM Na-ascorbate, 3 mM Na-pyruvate, 0.5 mM CaCl_2_, and 10 mM MgSO_4_. The pH of the solution was titrated to 7.3-7.4 with concentrated HCl. Coronal slices were cut at a thickness of 300 μm with using a linear slicer PRO7 (Dosaka EM, Kyoto, Japan). Slices were initially recovered for ≤ 15 min at 34°C and then transferred to a holding chamber containing HEPES holding aCSF (92 mM NaCl, 2.5 mM KCl, 1.25 mM NaH_2_PO_4_, 30 mM NaHCO_3_, 20 mM HEPES, 25 mM glucose, 2 mM thiourea, 5 mM Na-ascorbate, 3 mM Na-pyruvate, 2 mM CaCl_2_ and 2 mM MgSO_4_) at room temperature for at least 1 h before starting the recording. The slices were then transferred to the recording chamber mounted on a FN1 microscope (Nikon, Tokyo, Japan), in which they were submerged and perfused at a flow rate of 1-2 ml/min with recording aCSF containing the following: 124 mM NaCl, 5 mM KCl, 1.2 mM NaH_2_PO_4_, 1.5 mM MgCl_2_, 2.5 mM CaCl_2_, 10 mM glucose, 24 mM NaHCO_3_. All aCSF solutions were continuously bubbled with carbogen (95% O_2_; 5% CO_2_). Differential interference contrast (DIC) microscopy (C10639, Hamamatsu, Japan) was used to visualize pyramidal neurons in the PFC. Patch pipettes (5-6 MΩ) were prepared from borosilicate capillary glass using a micropipette puller PC-10 (NARISHIGE, Tokyo, Japan). Current clamp recordings were carried out at 32-33°C using a pipette filled with intracellular solution consisting of: 140 mM K-gluconate, 4 mM KCl, 10 mM HEPES, 0.2 mM EGTA, 4 mM MgATP, 0.3 mM Na_2_GTP, pH 7.3 with KOH in the presence of bicuculline (20 μM), CGP52422 (10 μM), and APV (50 μM) to block GABA_A_ and _B_ and NMDA receptors, respectively. Signals were recorded using a Multiclamp 700B amplifier (Molecular Devices, LLC, Orleans Drive Sunnyvale, CA), digitized using a Digidata 1440A interface board, filtered between 2 kHz, sampled at 10 kHz, and analyzed with pClamp10 software. Layer V PFC pyramidal neurons were recorded at holding potential of approximately –65 mV with constant current. A hyperpolarizing step (–20 pA) prior to each stimulation was used to monitor any changes in access resistance. Postsynaptic potentials were evoked by constant current single stimulation pulses (100 μs, 10-100 μA) delivered with a 20-s interstimulus interval using monopolar stimulating electrodes positioned in layer I.

**5-choice serial-reaction time task**

The experiment consists of two sessions, training and testing session. The training session was initiated with 7-week-old of male Long-Evans rats, and the testing session was conducted at 12-13 months old. Throughout the experimental period, food was restricted to 80-85% of their free-feeding body weight. The training and testing were conducted using four operant chambers enclosed in sound-attenuating boxes (Med Associates Inc., St Albans, VT). Each chamber contained a curved wall with five contiguous apertures. Food pellets were supplied automatically into a magazine located in the opposite wall of the five contiguous apertures in the chamber, and a photocell beam was used to detect head entries into the magazine. In the training session, a pellet was delivered into the magazine at the start of each session to facilitate initiation of the first trial. After a 5 s-intertrial interval (ITI), a light stimulus was presented in one of the five apertures followed by the 5 s-limited hold without light stimulus. Durations of light stimuli was set at 30 s and was gradually decreased during training to 2 s. Rats were required to nose-poke in the illuminated aperture. Correct responses (nose-poke responses in the illuminated aperture during a light stimulus and limited hold) resulted in the delivery of a food pellet into the magazine with sound and light signals above the magazine presented for 2 s. Incorrect responses (responses in non-illuminated apertures), omissions (failure to respond during the limited hold) and premature responses (responses occurring prior to the presentation of the stimulus) were punished by a 5 s-timeout periods with extinction of the house light and no delivery of food. Accuracy was calculated as the number of correct responses / (number of correct responses + number of incorrect responses) × 100. Each session lasted for 35 min or until 100 trials were completed. Rats were trained until they achieved criterion performance (>75% accuracy and <20 omissions) over 3 consecutive days. In the testing session, the duration of light stimuli on an aperture was set to 0.5 s with a variable ITI schedule (4, 5, 7, or 10 s). Equal numbers of each ITI were randomly presented over 100 trials. TAK-653 at 0.3 mg/kg or vehicle was orally administered 2 h prior to testing in a cross-over design. Data were indicated as the mean ± SEM of number of correct responses, omissions, and premature responses. For sub-population analyses, a median split of the population was undertaken based on their correct response scores under vehicle treatment to ensure no interaction with performance changes across the study ([Robinson, 2012](#_ENREF_6)).

**Radial arm maze test**

The experiment was performed using 9-week-old male Long-Evans rats as described previously (Zajaczkowski et al., 1996) with a minor modification. Each arm was 50 cm long, 10 cm wide and 40 cm high, and the maze was elevated 50 cm above the floor. After being fasted for 24 h, male Long-Evans rats were food-restricted to 85% of free-feeding body weight on the first day of exposure to the maze and throughout the experimental period. The experiment consists of two sessions, training and testing session. In the training session, rats were habituated to the maze and then trained. Reinforcement consisted of 3 food pellets (45 mg each) in the food cup was used. On the first day of habituation to the maze, reinforcements were placed near the entrance and at the mid-point of each arm. Three rats were placed on the maze at one time and allowed to explore and consume the pellets for 8 min. On the second day of habituation, each rat was placed on the maze and allowed 5 min to consume the pellets placed at the mid-point and in the food cup at the end of each arm. From the third day, reinforcement was placed in the food cup at the end of each arm. Rats were well trained to collect pellets placed on the edge of each arm. The learning criterion for the testing session was defined as 2 errors or less for two consecutive days. In the previous day of the testing session, baseline level of performance was qualified to confirm that rats completed collecting all pellets placed in the 8 arms with two errors or less. In the testing session, each rat was placed on the maze facing away from the experimenter and facing the fixed arm at the start of the trial. The entry of rats into each arm was recorded in sequence. Rats were allowed to explore until all pellets in the 8 arms were consumed, or 5 min had elapsed. Entry into an arm previously chosen was counted as an error. If an animal failed to choose all eight arms in 5 min, the number of unchosen was also counted as errors. TAK-653, LY451646 or AMPA was administered 1.5 h, 1 h or 0 h prior to the administration of vehicle or MK-801, respectively. Thirty min after dosing of vehicle or MK-801, rats were placed on the maze. Data were indicated as the mean ± SEM in the testing session.

**Delayed match-to-sample tasks**

The experiment was performed using 4-6 years old male cynomolgus monkeys (Macaca fascicularis) weighing 4-6 kg. Monkeys were maintained at 80% of free-feeding body weight throughout the experiment. Four monkeys were trained to perform DMTS task using a Cambridge Neuropsychological Test Automated Battery (CANTAB) system (CeNes, Cambridge, UK) (Weed et al., 1999). Briefly, a trial was initiated by presentation of an image of a sample object on the screen. The monkey had to touch this object on the screen within 30 s. Then, the sample object was diminished from the screen, and a variable delay (0, 4, 8, and 16 s) was ensured. After the delay, the sample object was re-presented together with three other objects. The monkey had to choose the sample object from the four objects and a collect choice was reinforced by the food reward. The ITI was 5 s and one session consisted of 96 trials (24 trials of each delay of 0, 4, 8, and 16 s). The variable delay durations were randomly presented within the 96 trials. The criterion for the experimental use of monkeys was 70% or more of the correct response in the 96 trials. In the testing session, TAK-653 (0.06 mg/kg) was administered orally to monkeys 6 h before DMTS testing. The correct responses were recorded for all trials during test sessions. Data were subdivided according to 4-s delay intervals consisting of each 24-trials of the session and represented as the mean ± SEM of % trials correct over 96 trials per session.

**Supplementary figures and tables**

**Figure S1.**

Fig. S1 Effect of PF-04958242 on Ca^2+^ influx in rat primary hippocampal neurons and cognitive functions. (A) Effects of PF-04958242 on Ca^2+^ influx in primary hippocampal neurons. PF-04958242 was applied in the presence or absence of 1 μM AMPA. Data are represented as mean ± SD (n = 4). (B) PF-04958242 (0.01, 0.1 and 1 mg/kg, p.o.) was administered to rats 0.5 h prior to the acquisition and the retention trials. Novelty discrimination index (NDI) data were presented as the mean ± SEM (n = 10). Significant difference from vehicle-treated group was indicated by ^#^*P* ≤ 0.05 (Two-tailed Williams’ test). (C) At 0.5 h before administration of vehicle or MK-801 (0.08 mg/kg, s.c.), PF-04958242 was administered to rats. Thirty minutes after dosing of MK-801, rats were placed on the maze, and then the entry into the arm was recorded. The mean errors were indicated as the mean ± SEM (n = 6-18). ^*^*P* ≤ 0.05 statistically significant compared with vehicle-vehicle group (by Student’s test).**Figure S2.**

Fig. S2. Effects of TAK-653 on the number of correct responses, omissions and premature responses in the 5CSRTT performance in rats.

TAK-653 at 0.3 mg/kg was orally administered 2 h prior to the trial. Correct responses (mean ± SEM, n = 13) have been defined as the total number of nose-pokes in the correct aperture within the limited hold (5 s) in whole population of rats (A) or in the poor performing rats (D). Omissions (mean ± SEM, n = 13) represent the total number of failures to make any nose-pokes during the limited hold (5 s) in whole population of rats (B) or in the poor performing rats (E). Premature responses represent number of responses occurring prior to stimulus presentation in whole population of rats (C) or in the poor performing rats (F). ^*^*P* ≤ 0.05 (crossover ANOVA for the difference from vehicle-treated group).

**Figure S3.**

Fig. S3. Effect of TAK-137 on AMPA-R-mediated EPSPs in prefrontal cortical slice. Application of 3 μM TAK-137 for 10 min potently enhanced the suprathreshold response with a significant increase in evoked spikes and EPSP duration (n = 6).

**Figure S4.**

Fig S4. Comparison between TAK-653, LY451646 and AMPA in the presence or absence of agonist. The result of LY451646 was cited from reference (Kunugi *et al*., 2018). Effects on intracellular Ca^2+^ level (A, C) and AMPA-R-mediated currents (B, D) were shown.

**Table S1.**

AMPA-R subunit selectivity of TAK-653.

EC_50_ values of TAK-653 were obtained in Ca^2+^ influx assay using CHO cells expressing GluA1-4i + TARPs γ2 or expressing GluA1-4o + TARPs γ2. Each estimated EC_50_ is from 8 dose response curves conducted in triplicate. Values represented the fold difference in EC_50_ value (EC_50_ GluA1i / EC_50_ each GluA).


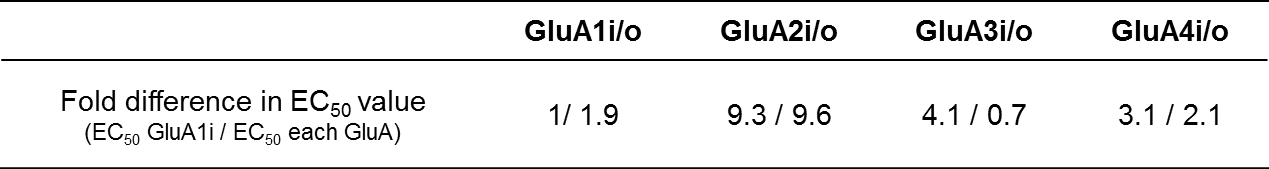


**Table S2.**

In vitro profile of TAK-653 (10 μM)


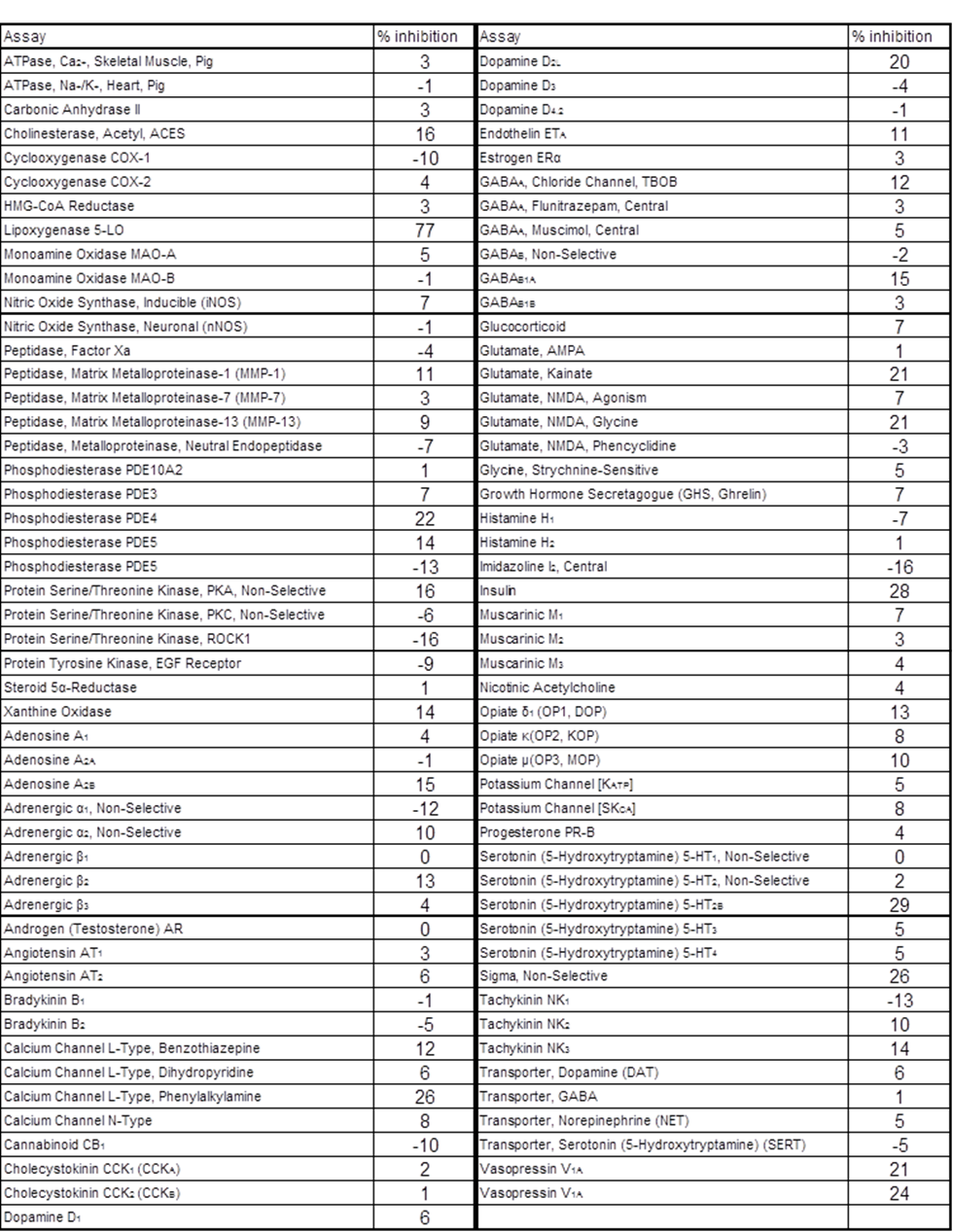


**Table S3.**

Plasma pharmacokinetic parameters of TAK-653

Mean (n = 3)


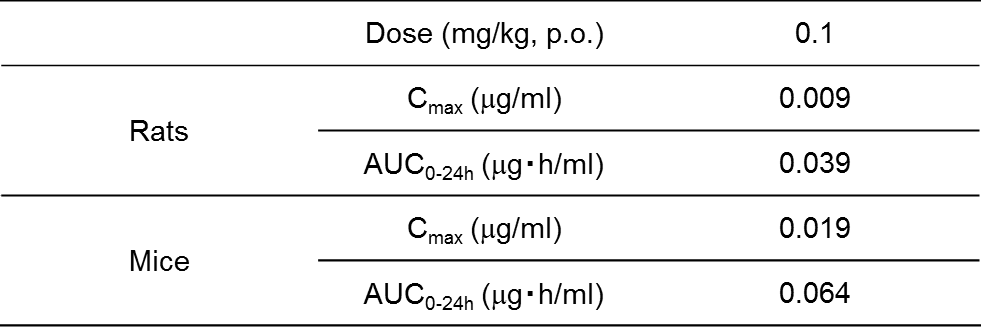


**Table S4.**

Rate of convulsions induced by TAK-653 (acute oral treatment) in rats.

(A) Acute effect on convulsions in safety pharmacology studies (n = 6). (B) Effect of first administration on convulsions in a thirteen-week oral gavage toxicity study (n = 10).


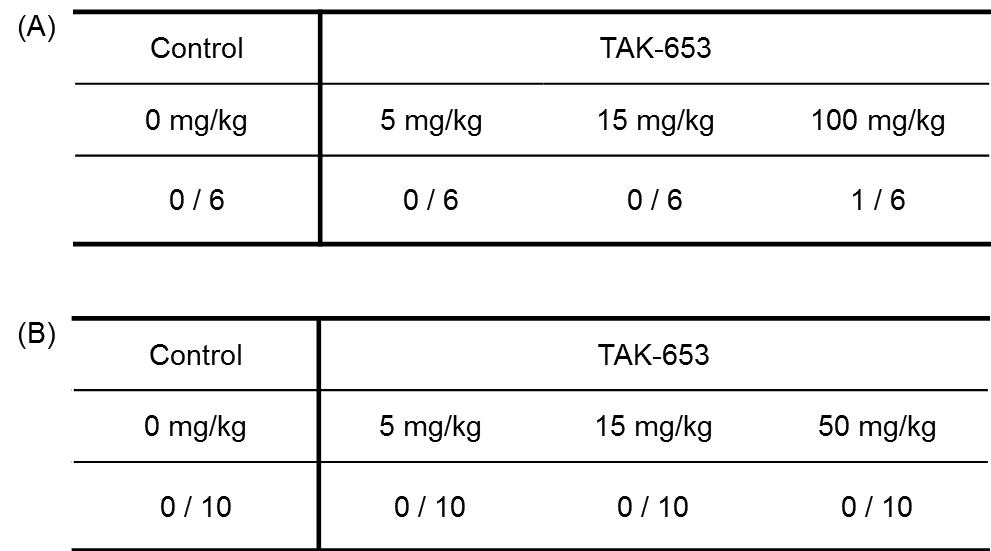


**Table S5.**

Single dose oral pharmacokinetic profile of TAK-653 in rats (n = 3).


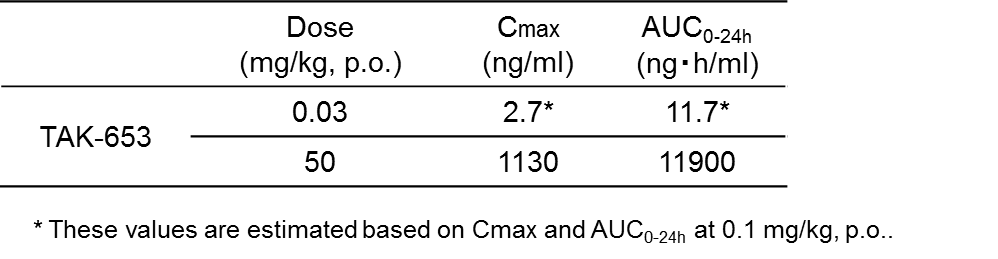


**Table S6.**

Data collection and refinement statistics

^a^R_sym_ = Σ_h_ Σ_i_ |I(h)_i_ – <I(h)>|/Σ_h_ Σ_i_ <I(h)>, where <I(h)> is the mean intensity of symmetry-related reflections. R_meas_ = Σ_h_ [N/(N-1)]^1/2^ Σ_i_ |I(h)i – <I(h)>|/Σ_h_ Σ_i_ <I(h)>, R_pim_ = Σ_h_ [1/(N-1)]^1/2^ Σ_i_ |I(h)_i_ – <I(h)>|/Σ_h_ Σ_i_ <I(h)>. ^b^CC_1/2_, Pearson correlation coefficient between independently merged halves of the data set. ^c^R_work_ = Σ ||F_obs_| – |F_calc_||/Σ |F_obs_|. R_free_ was calculated for randomly chosen 5% of reflections excluded from refinement. ^d^B-factor includes contributions from TLS parameters. ^e^Calculated with Coot. Values in parentheses are for the highest resolution shell.

**Table S7.**

Summary of AMPA ligands profile

|  | TAK-653 | TAK-137 ^[16, 20]^ | LY451646 ^[16, 20]^ | AMPA |
| --- | --- | --- | --- | --- |
| Binding affinity to the GluA2o LBD by SPA using [^3^H]-HBT1 | | | | |
| IC_50_ (μM) | 0.26 | 0.065 | 0.39 | nt |
| Intracellular Ca^2+^ level * | | | | |
| Potentiation EC_50_ (μM) | 0.93 | 0.42 | 0.78 | nt |
| Agonistic effect @30 μM | 4.8% | 7.6% | 88% | nt |
| AMPA-R currents ^#^ | | | | |
| Potentiation EC_50_ (μM) | 4.4 | 1.4 | 1.9 | nt |
| Agonistic effect @30 μM | 1.7% | 6.4% | 39% | nt |
| Exposure margin against seizure after acute treatment in rats (plasma C_max_ and AUC_plasma_) | | | | |
| Margin based on plasma C_max_ (fold) | 419 | 42 | 3.4 | nt |
| Margin based on AUC_plasma_ (fold) | 1017 | 122 | 4.0 | nt |
| Evoked AMPA-R-mediated EPSPs | | | | |
| Single effect on  Ca^2+^ level | ─ | ─ | ++ | + |
| Single effect on  AMPA-R currents | ─ | ─ | + | ++ |
| Synaptic EPSP response | ++ | ++ | + | ─ |
| Cognitive effects | | | | |
| NORT  (mg/kg, p.o.) | Effective  (0.03, 0.1, 0.3) | Effective  (0.1, 1) | Effective  (1, 3) | No effect |
| RAM  (mg/kg, p.o.) | Effective  (0.1, 0.3, 3, 10) | Effective  (0.2, 0.6) ^\^ | No effect | No effect |

nt: Not tested, -: No effect or slight effect, +: Potent, ++: Very potent

EC_50_ value was calculated from dose-response curve in the presence of 5 μM AMPA for intracellular Ca^2+^ levels or 1 μM AMPA for AMPA-R currents using a nonlinear regression.

^*^ % of 5 μM AMPA + 10 μM HBT1 response

^#^ % of AMPA (100 μM)-induced steady-state response

^\^ The plasma and brain concentrations of TAK-137 under fasted conditions were 67% and 71% of those under the fed conditions. Thus, 0.2 and/or 0.6 mg/kg of TAK-137 were used under fasted conditions.

**References** **(for Supplementary Information)**

Chen VB, Arendall WB, 3rd, Headd JJ, Keedy DA, Immormino RM, Kapral GJ*, et al* (2010). MolProbity: all-atom structure validation for macromolecular crystallography. *Acta Crystallogr D Biol Crystallogr* **66**(Pt 1): 12-21.

Emsley P, Lohkamp B, Scott WG, Cowtan K (2010). Features and development of Coot. *Acta Crystallogr D Biol Crystallogr* **66**(Pt 4): 486-501.

Kunugi A, Tajima Y, Kuno H, Sogabe S, Kimura H (2018). HBT1, a Novel AMPA Receptor Potentiator with Lower Agonistic Effect, Avoided Bell-Shaped Response in In Vitro BDNF Production. *J Pharmacol Exp Ther* **364**(3): 377-389.

Murshudov GN, Skubak P, Lebedev AA, Pannu NS, Steiner RA, Nicholls RA*, et al* (2011). REFMAC5 for the refinement of macromolecular crystal structures. *Acta Crystallogr D Biol Crystallogr* **67**(Pt 4): 355-367.

Otwinowski Z, Minor W (1997). Processing of X-ray Diffraction Data Collected in Oscillation Mode *Methods in Enzymology* **276**: 307-326.

Robinson ES (2012). Blockade of noradrenaline re-uptake sites improves accuracy and impulse control in rats performing a five-choice serial reaction time tasks. *Psychopharmacology (Berl)* **219**(2): 303-312.

Vagin A, Teplyakov A (2010). Molecular replacement with MOLREP. *Acta Crystallogr D Biol Crystallogr* **66**(Pt 1): 22-25.

Winn MD, Ballard CC, Cowtan KD, Dodson EJ, Emsley P, Evans PR*, et al* (2011). Overview of the CCP4 suite and current developments. *Acta Crystallogr D Biol Crystallogr* **67**(Pt 4): 235-242.
